# NLRP3 signalling in NSCLC: distinguishing expression, inflammasome activation and context-dependent function

**DOI:** 10.3389/fimmu.2026.1983973

**Published:** 2026-10-20

**Authors:** Xin Wang, Yangli Zhong, Bin Pan, Kai Yang, Hongmei Tan, Ling Huang, Xi Li

**Affiliations:** 1 School of Clinical Medicine, Chengdu Medical College, Chengdu, Sichuan, China; 2 Department of Respiratory and Critical Care Medicine, The First Affiliated Hospital of Chengdu Medical College, Chengdu, Sichuan, China; 3 Department of Dermatology, The First Affiliated Hospital of Chengdu Medical College, Chengdu, Sichuan, China; 4 Key Laboratory of Geriatric Respiratory Diseases of Sichuan Higher Education Institutes, Chengdu, Sichuan, China; 5 Department of Pharmacy, The First Affiliated Hospital of Chengdu Medical College, Chengdu, Sichuan, China; 6 Department of Integrated Traditional Chinese and Western Medicine, Clinical Medical College and The First Affiliated Hospital of Chengdu Medical College, Chengdu, China

**Keywords:** immune checkpoint blockade, inflammasome activation, mechanistic evidence classification, NLRP3 inflammasome, non-small cell lung cancer, pyroptosis, tumour microenvironment

## Abstract

Non-small cell lung cancer (NSCLC) remains a major cause of cancer-related mortality despite advances in targeted therapy and immune checkpoint blockade. NLRP3-associated inflammation has attracted increasing interest in NSCLC, yet interpretation of the available evidence remains challenging because molecular expression, inflammasome activation, cytokine processing, pyroptotic execution and pathway dependence are frequently considered together. Here, we present a structured narrative review and an evidence framework that separates the biological event directly measured from the level of NLRP3-specific mechanistic support, while independently considering cellular source, disease relevance and treatment context. Current evidence indicates that NLRP3-specific mechanistic dependence in NSCLC has been demonstrated only in selected experimental settings, particularly treatment-conditioned macrophage models. Treatment-naïve human evidence reveals compartment-specific inflammasome-associated responses in alveolar macrophages and peripheral leukocytes but does not establish NLRP3 dependence. Tumour-cell studies support treatment-induced inflammasome- and pyroptosis-associated phenotypes, although causal attribution to NLRP3 is often incomplete. Single-cell and spatial studies mainly identify NLRP3-related transcriptional states or candidate microenvironmental niches and cannot, by themselves, demonstrate functional inflammasome activation or NLRP3-dependent interactions. Clinical trials targeting IL-1β provide evidence for downstream inflammatory modulation but do not directly evaluate NLRP3 inhibition. Overall, current evidence supports context-dependent NLRP3-associated biology in NSCLC rather than a uniform biomarker or therapeutic target. Future studies integrating cell-resolved localisation, activation-specific measurements, pathway perturbation and longitudinal validation are required to determine whether clinically relevant NLRP3-dependent states can be reproducibly identified.

## 1 Introduction

Non-small cell lung cancer (NSCLC) accounts for the majority of lung cancer cases and remains a major cause of cancer-related mortality worldwide (1, 2). Although molecularly targeted therapies and immune checkpoint blockade (ICB) have substantially improved outcomes in selected patient populations, durable clinical benefit can be limited by primary and acquired resistance and by marked interpatient and intratumoural heterogeneity (3, 4). NSCLC is increasingly understood as a multicellular ecosystem in which malignant epithelial cells interact with myeloid populations, lymphocytes, fibroblasts, endothelial cells, and other stromal compartments to shape inflammatory and immune-regulatory states within the tumour microenvironment (5, 6).

The NLRP3 inflammasome is a cytosolic multiprotein signalling platform that links cellular stress to inflammatory cytokine processing and, in some settings, pyroptotic cell death (7). Canonical NLRP3 activation is generally described as a priming phase followed by inflammasome assembly and downstream effector activation (7, 8). Priming increases the expression of NLRP3 and pro-IL-1β, often through NF-κB-dependent signalling, whereas subsequent activating signals promote NLRP3 oligomerisation, ASC recruitment and speck formation, and caspase-1 activation (7, 8). Activated caspase-1 processes pro-IL-1β and pro-IL-18 into their mature forms and can cleave gasdermin D (GSDMD), generating the pore-forming GSDMD-N fragment and, when sufficiently engaged, pyroptotic membrane permeabilisation (7, 8). These events are mechanistically linked but experimentally distinct (8).

This distinction is particularly important in NSCLC because the available literature spans transcriptomic and spatial profiling, protein-based measurements, cytokine analyses, pharmacologically perturbed systems and genetically manipulated experimental models (9, 10). These approaches differ substantially in their ability to demonstrate productive inflammasome activation and NLRP3-specific pathway dependence, as activation-associated molecular events and causal pathway attribution require distinct experimental evidence (9, 11). Increased expression of NLRP3 or IL1B primarily reflects transcriptional priming or the availability of inflammasome components, rather than definitive inflammasome activation. In contrast, evidence of inflammasome assembly, caspase-1 activation, maturation of IL-1β/IL-18, and GSDMD cleavage provides direct readouts of different stages of functional inflammasome engagement and downstream pyroptotic execution (11, 12). None of these readouts, when interpreted in isolation, is sufficient to establish NLRP3-specific causality, because comparable inflammatory cytokine maturation and pyroptotic phenotypes can also result from activation of other inflammasome sensors or inflammatory caspase-dependent pathways (11, 13).

The involvement of inflammasome signalling in cancer and lung cancer has been reviewed previously (14). Cancer-focused reviews have highlighted the context-dependent and sometimes opposing roles of NLRP3 in tumour biology, immune regulation, and responses to anticancer therapies (15). Importantly, da Costa et al. specifically reviewed inflammasome-driven inflammation across lung-cancer initiation, progression, metastasis and therapy resistance, with particular emphasis on NSCLC (16). Thus, the link between NLRP3/inflammasome signalling and lung cancer is an established concept that has been discussed in previous reviews (15, 16). Rather, the present structured narrative review addresses a different interpretative challenge: heterogeneous experimental approaches are frequently interpreted together despite capturing distinct biological events, levels of pathway activation, mechanistic dependencies, and implications for disease relevance (11). Previous lung-cancer and cancer-wide reviews have provided important biological and mechanistic syntheses of inflammasome-related functions (15, 16); however, an experiment-level evidence framework that systematically separates biological readouts, NLRP3-specific mechanistic attribution, and evidence context remains insufficiently developed in NSCLC. A direct comparison of the scope, organising principles, and evidence-classification features of previous lung-cancer and cancer-wide reviews and the present review is provided in **Supplementary Table S3** (14, 16).

Accordingly, the incremental contribution of this review is primarily organisational and interpretative. Individual experimental evidence components were classified according to the biological event directly measured and the strength of NLRP3-specific mechanistic attribution, whereas evidence source, cellular compartment, and treatment context were independently annotated (11, 13). This approach is intended to distinguish descriptive associations from functional pathway engagement and to avoid extrapolating mechanistic conclusions from one experimental layer to correlative human observations or context-dependent spatial measurements (11).

We therefore apply a two-axis evidence framework throughout this review to systematically organise experimental evidence and interpret the strength of NLRP3-specific mechanistic inference. The A-axis describes the biological event directly assessed, whereas the B-axis describes mechanistic attribution to NLRP3. Evidence source is classified independently as Direct NSCLC evidence, Indirect NSCLC evidence, Mechanistic extrapolation or Clinical downstream-intervention evidence. These dimensions are descriptive rather than hierarchical, and evidence-source categories indicate disease and experimental context rather than mechanistic strength. The A1–A5 and B1–B3 labels are author-proposed operational categories for evidence organisation and do not represent established consensus nomenclature. **Figure 1** maps NLRP3-associated states across cellular, spatial, and treatment contexts, whereas **Figure 2** presents the two-axis framework and accompanying evidence-source classification. **Table 1** provides the operational definitions, and **Supplementary Table S1** applies them at the level of individual experimental evidence components.

**Figure 1:**
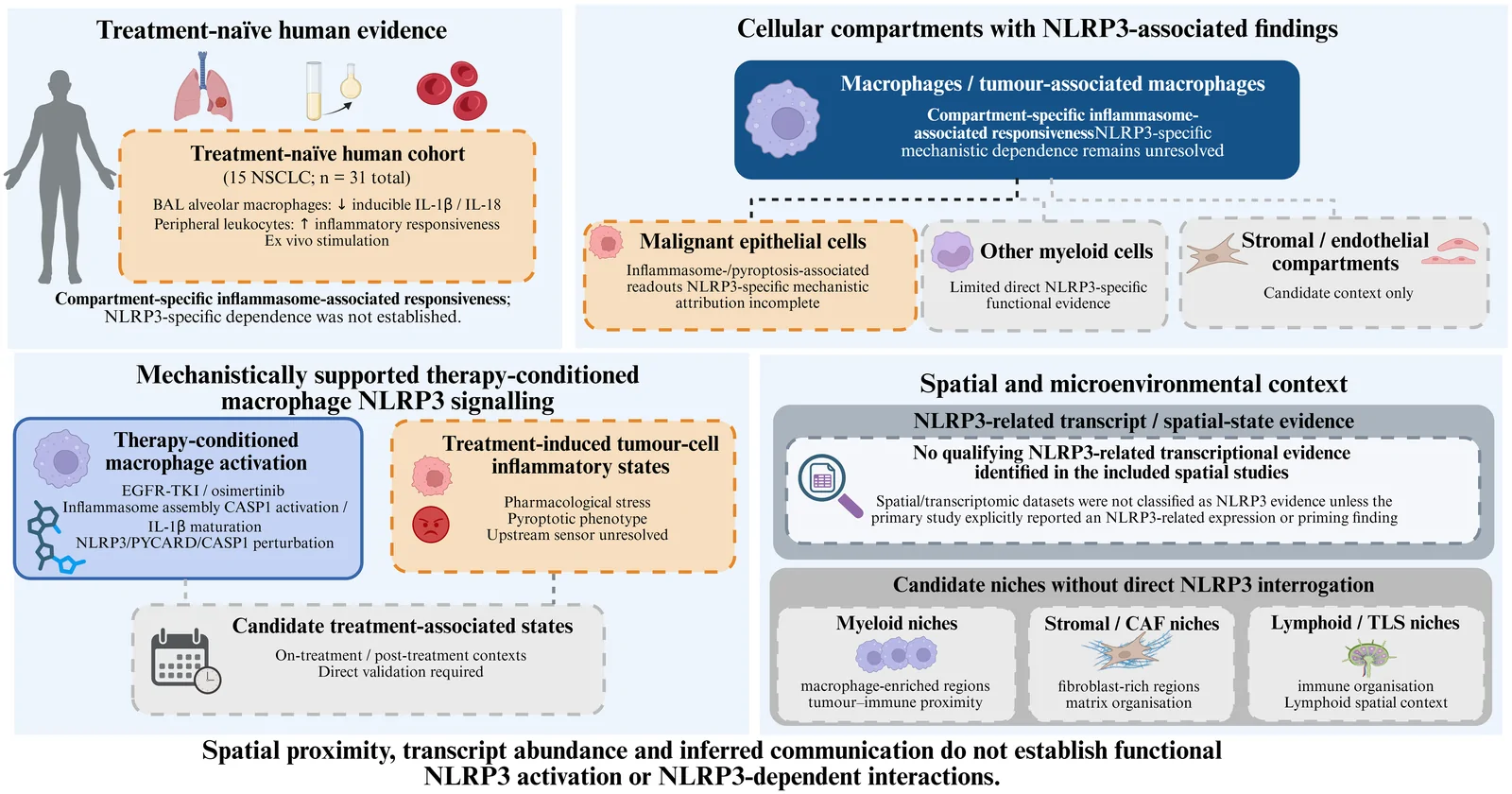
Evidence-stratified map of NLRP3-associated findings in NSCLC. E Evidence is separated into mechanistically supported NLRP3-dependent findings, NLRP3-associated observations with incomplete mechanistic attribution, NLRP3-related transcript/spatial-state evidence, and candidate contextual niches without direct NLRP3 interrogation. Treatment-naïve human evidence demonstrates compartment-specific inflammasome-associated responsiveness but does not establish NLRP3-specific dependence. Mechanistically supported canonical NLRP3 signalling is confined to selected treatment-conditioned macrophage models, whereas tumour-cell studies generally show incomplete NLRP3-specific mechanistic attribution. No spatially resolved study included in the present evidence map met the predefined criteria for NLRP3-related transcriptional evidence; instead, current single-cell and spatial studies primarily define candidate cellular states and microenvironmental contexts for subsequent functional investigation. Spatial proximity, transcript abundance and inferred communication do not, by themselves, establish functional NLRP3 activation, pathway dependence or interaction directionality. Dashed connectors indicate contextual or hypothesis-generating relationships and do not denote established causal directionality or evidentiary hierarchy. Created in BioRender. xin, W. (2026) https://BioRender.com/k2ew3ps.

**Figure 2:**
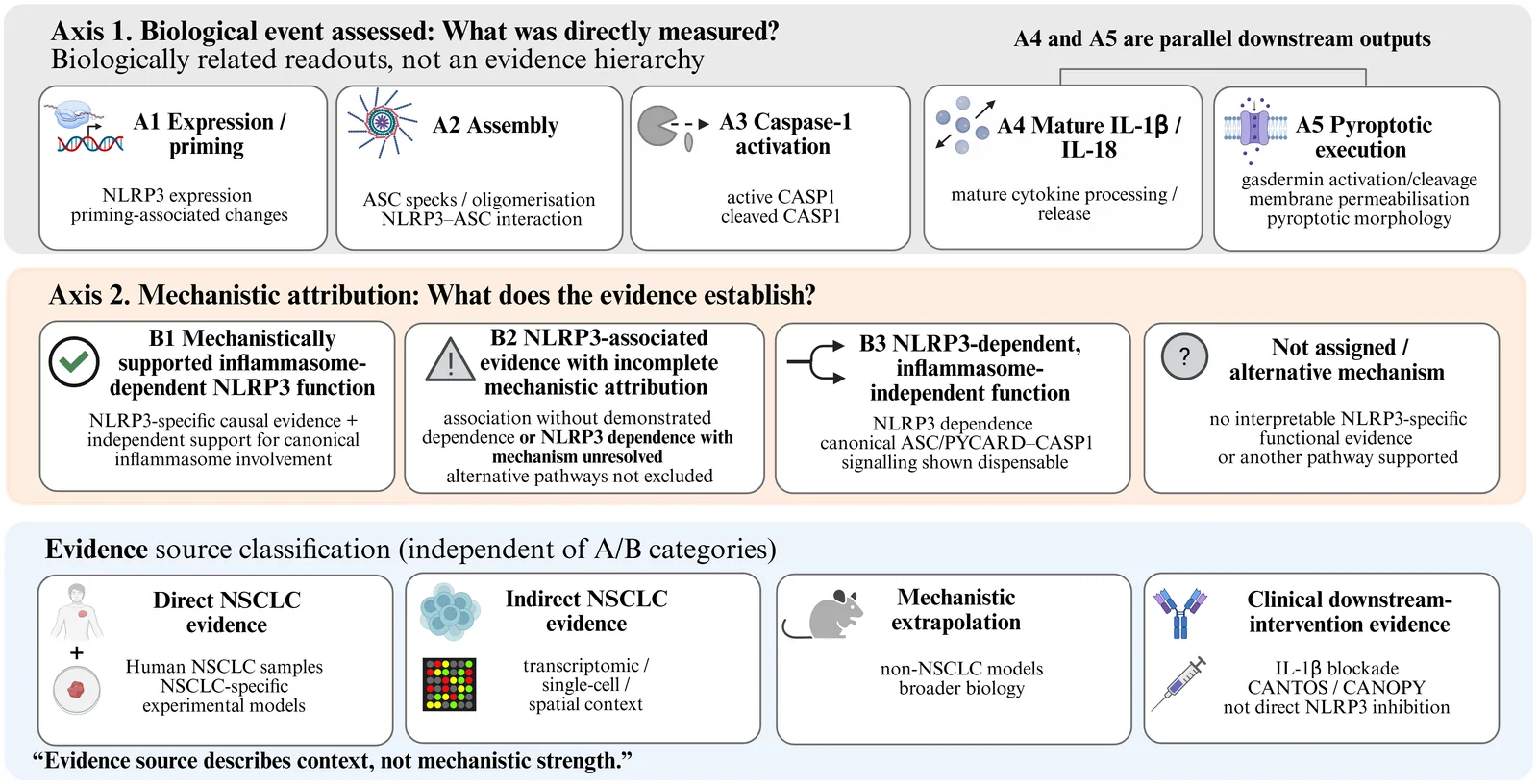
Evidence-based framework for interpreting NLRP3-associated findings in NSCLC. The framework separates the biological event directly assessed (A-axis) from mechanistic attribution to NLRP3 (B-axis), with evidence source classified independently. The A-axis includes expression/priming, inflammasome assembly, caspase-1 activation, mature IL-1β/IL-18 production and gasdermin-mediated pyroptotic execution. These categories represent distinct biological readouts rather than hierarchical levels of evidentiary strength, and cytokine maturation and pyroptotic execution may occur as parallel downstream outcomes. The B-axis distinguishes mechanistically supported inflammasome-dependent NLRP3 function (B1), NLRP3-associated evidence with incomplete mechanistic attribution (B2), NLRP3-dependent inflammasome-independent function (B3), and not assigned/alternative mechanisms. B1 requires NLRP3-specific causal evidence together with independent evidence supporting canonical inflammasome involvement. ASC/PYCARD or CASP1 functional validation represents the most direct evidence but is not mandatory; where unavailable, convergent activation-specific evidence, such as inflammasome assembly together with caspase-1-dependent cytokine maturation, may provide independent support. NLRP3 perturbation alone, downstream inflammatory readouts alone, or pharmacological inhibition alone are insufficient for B1 assignment. B3 requires pathway-separation evidence demonstrating that canonical NLRP3–ASC/PYCARD–CASP1 signalling is dispensable. Evidence-source categories describe disease and experimental context rather than mechanistic strength, and classification is applied to individual experimental evidence components rather than entire publications. Created in BioRender. xin, W. (2026) https://BioRender.com/k2ew3ps.

**Table 1:** Evidence classification framework for interpreting NLRP3-associated findings in NSCLC.

Dimension	Category	Definition	Operational criteria and interpretation
**Axis 1: Biological event assessed (A-axis)**	**A1. NLRP3-related expression or priming-associated state**	Altered NLRP3 abundance or inflammatory priming without direct evidence of productive inflammasome activation.	Increased NLRP3 mRNA or protein, enhanced pro-IL-1β expression, or NF-κB-associated priming or related transcriptional changes supports A1. A1 indicates pathway availability or priming but does not establish inflammasome assembly, caspase-1 activation or productive inflammasome signalling (7, 9, 11, 12, 32).
	**A2. Inflammasome assembly-related event**	Direct evidence of recruitment, oligomerisation or assembly of inflammasome components.	ASC/PYCARD speck formation or oligomerisation, experimentally demonstrated NLRP3–ASC interaction, or comparable assembly-related evidence supports A2. NLRP3 expression alone does not establish inflammasome assembly (7, 8, 18, 83).
	**A3. Caspase-1 activation**	Evidence of inflammatory caspase-1 activation.	Cleaved/processed CASP1 and/or a validated caspase-1 activity assay supports A3. CASP1 activation is not specific to NLRP3 and therefore does not by itself identify NLRP3 as the upstream inflammasome sensor (7–9, 18).
	**A4. Mature IL-1β/IL-18 production**	Evidence of processing and/or release of mature inflammasome-associated cytokines.	Detection of mature or processed IL-1β and/or IL-18 supports A4. Increased total transcript or precursor protein alone is insufficient to demonstrate cytokine maturation. Mature IL-1β/IL-18 supports inflammasome-associated cytokine processing but does not identify NLRP3 as the upstream sensor, because alternative inflammasomes and inflammatory caspase-dependent pathways may produce similar downstream outputs (7, 9, 12, 18).
	**A5. Gasdermin-mediated pyroptotic execution**	Evidence of gasdermin activation associated with membrane permeabilisation or an experimentally supported pyroptotic phenotype.	Gasdermin cleavage/activation together with compatible membrane permeabilisation or pyroptotic morphology supports A5. LDH release or other nonspecific lytic-cell-death readouts alone are insufficient. GSDMD cleavage is compatible with inflammatory-caspase-driven pyroptotic execution but is not specific to canonical NLRP3–CASP1 signalling, because GSDMD may also be activated downstream of non-canonical inflammatory caspases. Other gasdermins, including GSDME, should be interpreted according to their experimentally demonstrated upstream protease and signalling context (17, 19, 78, 91).
**Axis 2: Mechanistic attribution (B-axis)**	**B1. Mechanistically supported inflammasome-dependent NLRP3 function**	Evidence that the phenotype depends causally on NLRP3, with independent evidence supporting canonical inflammasome involvement.	Minimum requirement: NLRP3-specific causal perturbation must alter the phenotype of interest, together with independent evidence that the relevant phenotype or downstream response depends on canonical inflammasome signalling. Functional perturbation of ASC/PYCARD and/or CASP1 provides the strongest pathway-separation evidence. Where such perturbation is not technically available, convergent activation-specific evidence may support B1 only when it directly links NLRP3 perturbation to inflammasome assembly and caspase-1-dependent downstream processing. NLRP3 perturbation alone, downstream inflammatory readouts alone, or pharmacological inhibition without independent target validation are insufficient for B1 assignment (7, 18, 23, 83, 88).
	**B2. NLRP3-associated evidence with incomplete mechanistic attribution**	Evidence associated with NLRP3 or inflammasome-related biology in which NLRP3 dependence and/or canonical inflammasome involvement is not causally established.	Includes expression- or correlation-based findings without demonstrated NLRP3 dependence; inflammasome-associated downstream readouts, including CASP1 activation, mature IL-1β/IL-18 production or gasdermin cleavage, without NLRP3-specific causal validation; pharmacological effects without adequate target-specific validation; and NLRP3-dependent phenotypes in which canonical inflammasome-dependent versus inflammasome-independent mechanisms remain unresolved. Where plausible alternative inflammasomes or non-canonical inflammatory pathways have not been experimentally distinguished, classification remains B2 (7, 18, 20, 21).
	**B3. NLRP3-dependent, inflammasome-independent function**	Evidence that NLRP3 causally contributes to a phenotype while canonical NLRP3–ASC/PYCARD–CASP1 signalling is dispensable.	Requires an NLRP3-specific perturbation that alters the phenotype together with positive pathway-separation evidence demonstrating that canonical NLRP3–ASC/PYCARD–CASP1 signalling is dispensable. Absence of ASC/PYCARD, CASP1, cytokine maturation or pyroptotic readouts alone is insufficient for B3 assignment; the phenotype must be experimentally separated from canonical inflammasome signalling (20, 21).
	**Not assigned/alternative mechanism**	Findings for which NLRP3-specific mechanistic attribution cannot be made, or for which another inflammatory mechanism is experimentally supported.	Not assigned is used when available evidence is insufficient for defensible assignment to B1–B3. Alternative mechanism is used when experimental evidence supports another inflammasome, a non-canonical inflammatory caspase or another inflammatory pathway. A plausible but untested competing pathway does not by itself exclude B2 (18, 20, 21).
**Evidence-source classification**	**NSCLC-specific evidence**	Evidence generated from human NSCLC material or NSCLC-specific experimental models.	Includes untreated or treatment-exposed human NSCLC samples, ex vivo patient-derived material, NSCLC-derived cell lines, xenografts, and NSCLC-relevant syngeneic or genetically defined models. Species, treatment status, cellular source and model type should be reported explicitly. This designation describes disease and experimental context rather than mechanistic strength.
	**NSCLC contextual evidence**	NSCLC-specific contextual evidence without direct demonstration of functional NLRP3 activity.	Includes transcriptomic, single-cell, spatial-transcriptomic or multiplex-imaging studies that define relevant cellular states, tissue niches or microenvironmental architecture but do not directly establish inflammasome assembly, CASP1 activation, cytokine maturation or NLRP3-dependent function (22, 61, 63, 68, 70, 71, 80, 81).
	**Mechanistic extrapolation**	Evidence generated outside NSCLC that informs biological plausibility or mechanistic interpretation.	Includes structural, biochemical, immunological or cell-biological studies performed in non-NSCLC systems. Such evidence may support mechanistic interpretation but should not be treated as direct evidence of NLRP3 function in NSCLC.
	**Clinical downstream-intervention evidence**	Human interventional evidence targeting downstream inflammatory mediators without directly testing NLRP3 inhibition or NLRP3 dependence.	Includes IL-1β-targeted studies such as the CANTOS lung-cancer analysis and CANOPY trials. These studies inform the clinical consequences of downstream IL-1β modulation but should not be interpreted as direct clinical tests of NLRP3 inhibition or NLRP3 pathway dependence (24–28).

The molecular events underlying A1–A5 are derived from established inflammasome and pyroptosis biology, including transcriptional priming, inflammasome assembly, caspase-1 activation, cytokine maturation and gasdermin-mediated pyroptotic execution (7–9, 11–13, 17–19, 32, 78, 83, 91). In contrast, the A1–A5 labels, B1–B3 mechanistic-attribution categories, minimum assignment criteria and evidence-source categories were developed specifically for this review and do not represent established consensus nomenclature. Bold text indicates the predefined evidence categories and corresponding classification codes (A1–A5 and B1–B3) used in the NLRP3 evidence framework.

## 2 Evidence framework and literature scope

Published studies have examined NLRP3-related processes across diverse experimental contexts, including untreated patient specimens, treatment-exposed models, malignant epithelial systems, immune compartments, and spatially resolved tumour microenvironments (15, 16). However, these approaches capture distinct aspects of NLRP3 biology and provide unequal levels of evidence for inflammasome activation and NLRP3-specific pathway dependence (11).

For example, increased NLRP3 expression may indicate inflammatory priming or increased pathway availability, whereas inflammasome assembly, caspase-1 activation, mature IL-1β/IL-18 production, and gasdermin-mediated pyroptotic execution represent mechanistically related but experimentally distinct biological events that require separate experimental assessment (11, 17). Likewise, transcriptomic and spatial studies may identify NLRP3-associated cellular states or candidate inflammatory niches, but these approaches alone cannot establish functional NLRP3 inflammasome activation (11).

To address these differences, this structured narrative review applies an author-defined evidence framework that separates three aspects of NLRP3-associated findings: (i) the biological event directly assessed, (ii) the strength of mechanistic attribution to NLRP3, and (iii) the disease and experimental context of the evidence source. These dimensions are considered independently to distinguish descriptive associations from functional pathway engagement and to evaluate the strength of NLRP3-specific mechanistic attribution (11, 13).

### 2.1 Literature search and evidence selection

The literature discussed in this review was identified through searches of PubMed, Web of Science and Europe PMC from database inception to August 20, 2026. The core search combined NLRP3/inflammasome terms with lung-cancer terms and pathway-related readouts. Because this NLRP3-focused strategy would not necessarily retrieve studies providing relevant spatial context without directly interrogating NLRP3, or clinical trials targeting downstream IL-1β, two supplementary topic-search routes were applied. First, NSCLC/lung-cancer terms were combined with single-cell, spatial-transcriptomic, multiplex-imaging and tumour-microenvironment terms to identify contextual cellular and spatial studies. Second, IL-1β, canakinumab, CANTOS and CANOPY terms were searched separately to identify downstream-intervention studies. Backward and forward citation tracking of eligible primary studies and relevant reviews was additionally used to identify potentially eligible studies not retrieved through the database searches. Database-specific core and supplementary search routes, including the terms used for each route, are provided in **Supplementary Table S2**.

The search was restricted to English-language publications. Primary human and experimental NSCLC/lung-cancer studies were retained when they informed the predefined evidence domains: NLRP3-related expression or priming, inflammasome assembly, caspase-1 activation, mature IL-1β/IL-18 production, gasdermin-mediated pyroptosis, NLRP3-dependent phenotypes, or NSCLC-specific cellular or spatial context. These biological domains reflect established features of inflammasome activation and pyroptotic execution (11, 17). Spatial, single-cell, transcriptomic or multiplex-imaging studies without direct functional NLRP3 assessment were retained only when they provided NSCLC-specific contextual evidence relevant to the predefined spatial evidence domain. Where the primary study did not directly report an NLRP3-related finding, the study was classified as candidate contextual evidence rather than NLRP3 evidence. Downstream IL-1β intervention trials were identified through the separate clinical-intervention search route and classified as clinical downstream-intervention evidence. Reviews were used for background and citation tracking rather than entered as primary evidence-map components.

For each eligible primary study, evidence was mapped at the level of individual experimental components rather than entire publications. Extracted fields included species/model/population, cellular source, treatment or stimulus, key readouts, perturbations, sample size or biological replication where reported, A/B framework classification, evidence-source category and principal limitations. **Supplementary Table S1** therefore separates mechanistic, observational, *in vivo* and contextual components when they support different inferences and includes supportive, null, contrary, compartment-specific, treatment-associated and context-dependent findings identified within the predefined scope.

This work was conducted as a structured narrative review rather than a formal systematic review. Accordingly, quantitative meta-analysis and formal risk-of-bias assessment were not undertaken. However, within the predefined eligibility scope, **Supplementary Table S1** was constructed to include all primary studies identified through the database searches, supplementary topic searches and citation-tracking procedures that met the stated inclusion criteria. Primary studies cited solely for general biological, clinical, technological or methodological background were not included in the formal evidence map. **Supplementary Table S2** documents the search routes used, while **Supplementary Table S1** provides the corresponding component-level evidence map.

### 2.2 Principles for evidence classification

Because NLRP3-associated observations encompass distinct biological events and different degrees of mechanistic resolution, we applied a two-axis framework to organise the evidence (**Figure 2**) (11). The A-axis describes the biological event directly assessed, whereas the B-axis describes mechanistic attribution to NLRP3. These dimensions are independent: A categories indicate what was measured, whereas B categories indicate how specifically the phenotype under investigation can be attributed to NLRP3. Classification is therefore applied to individual experimental evidence components rather than entire publications (11). The A1–A5 and B1–B3 labels and their operational assignment rules are proposed for this review and should not be interpreted as established consensus nomenclature.

The A-axis comprises five operational categories describing experimentally distinct biological readouts: A1, NLRP3-related expression or priming-associated state; A2, inflammasome assembly-related events; A3, caspase-1 activation; A4, mature IL-1β/IL-18 production; and A5, gasdermin-mediated pyroptotic execution. These categories are based on established features of inflammasome activation and pyroptotic execution (11, 17). These categories should not be interpreted as a sequential pathway or as increasing levels of evidentiary strength. In particular, caspase-1 activation, mature IL-1β/IL-18 production and gasdermin-mediated cell death do not, by themselves, identify NLRP3 as the upstream inflammasome sensor (18). A4 and A5 may occur as parallel downstream outcomes (17). Where pyroptotic execution is assessed, the gasdermin involved should be specified when experimentally determined because different gasdermins may reflect distinct upstream mechanisms (19). Detailed operational criteria for A1–A5 assignment are provided in **Table 1**.

The B-axis addresses NLRP3-specific mechanistic attribution. B1, mechanistically supported inflammasome-dependent NLRP3 function, requires two complementary components: (i) NLRP3-specific causal evidence demonstrating that perturbation of NLRP3 alters the phenotype of interest and (ii) independent evidence supporting canonical inflammasome involvement. Functional perturbation of ASC/PYCARD or CASP1 provides the most direct evidence for canonical inflammasome dependence but is not an absolute requirement. Where such perturbation is unavailable, convergent activation-specific evidence, such as inflammasome assembly together with caspase-1-dependent cytokine maturation, may provide independent support (7, 18). NLRP3 perturbation alone, downstream inflammatory readouts alone, or pharmacological inhibition alone are insufficient for B1 assignment. Genetic loss-of-function approaches can support NLRP3-specific causal attribution, whereas pharmacological inhibition requires independent support for target specificity.

B2, NLRP3-associated evidence with incomplete mechanistic attribution, includes both findings associated with NLRP3 without demonstrated NLRP3 dependence and NLRP3-dependent phenotypes for which canonical inflammasome-dependent versus inflammasome-independent mechanisms remain unresolved. Expression-based findings, correlative human observations, inflammasome-associated downstream readouts without NLRP3-specific causal validation and pharmacological effects without adequate target-specific validation therefore remain B2. Where alternative inflammasomes or non-canonical inflammatory caspases remain plausible but have not been experimentally distinguished, mechanistic attribution also remains B2.

B3, NLRP3-dependent, inflammasome-independent function, is assigned to phenotypes in which NLRP3-specific perturbation alters the phenotype and pathway-separation evidence demonstrates that canonical NLRP3–ASC/PYCARD–CASP1 signalling is dispensable (20, 21). Depending on the biological claim, assessment of ASC/PYCARD, CASP1, mature IL-1β/IL-18 signalling or gasdermin-mediated pyroptotic execution may provide additional support. Absence of a single downstream inflammasome readout is insufficient for B3 assignment.

Findings lacking an interpretable NLRP3-specific experimental or association-level component are annotated as not assigned. This includes contextual transcriptional, spatial or cellular-state observations in which functional NLRP3 activity is not directly interrogated. By contrast, findings for which another inflammasome, non-canonical inflammatory caspase or alternative inflammatory pathway is experimentally supported are annotated as alternative mechanism. Thus, an alternative pathway that remains plausible but untested does not by itself exclude B2 assignment, whereas experimentally supported attribution to another pathway favours classification as alternative mechanism.

Evidence source is annotated independently of the A/B framework and describes disease and experimental context rather than mechanistic strength. Direct NSCLC evidence refers to findings obtained from human NSCLC material or NSCLC-specific experimental models; this designation does not by itself imply functional inflammasome activation or NLRP3-specific causality. Indirect NSCLC evidence refers to transcriptomic, single-cell, spatial or cellular-state studies that define NSCLC-specific biological contexts without directly demonstrating functional NLRP3 activity (22). Mechanistic extrapolation refers to evidence generated outside NSCLC that informs biological plausibility or mechanistic interpretation but requires validation in an appropriate NSCLC setting. Clinical downstream-intervention evidence refers to human intervention studies targeting downstream inflammatory mediators associated with NLRP3 biology, without directly testing NLRP3 inhibition or NLRP3 dependence (23). This category includes IL-1β-targeted clinical studies such as the CANTOS lung-cancer analysis and the CANOPY trials (24–28).

These evidence-source categories are descriptive rather than hierarchical and should be interpreted together with species, cellular source, treatment context and experimental design (29). Detailed operational definitions are provided in **Table 1**, and their application to individual evidence components is summarised in **Supplementary Table S1**.

## 3 Conditions associated with NLRP3 inflammasome activation or activation competence in NSCLC

Mechanistically resolved evidence for NLRP3-specific pathway dependence in NSCLC has mainly been obtained under conditions of substantial cellular or treatment-related stress (7, 30). In contrast, treatment-naïve human evidence indicates compartment-specific inflammasome-associated responses in alveolar macrophages and peripheral leukocytes but does not establish NLRP3-specific causal dependence (31). Recurrent upstream events include lysosomal dysfunction, ionic and Ca²^+^ disturbance, mitochondrial injury, oxidative stress, metabolic perturbation, cytosolic DNA sensing, and treatment-induced damage (32, 33). These processes are highly interconnected. Lysosomal Ca²^+^ release can disrupt mitochondrial homeostasis, whereas mitochondrial injury can generate reactive oxygen species (ROS) and release mitochondrial DNA (mtDNA), thereby linking organelle stress to innate DNA-sensing pathways (30, 34).

### 3.1 Organelle stress: lysosomal dysfunction, Ca²^+^ dysregulation and mitochondrial injury

Mitochondrial dysfunction and lysosomal damage represent well-characterised upstream stress signals capable of promoting NLRP3 inflammasome activation; however, their presence alone does not establish NLRP3-specific inflammasome activation in NSCLC models (11). Loss of mitochondrial homeostasis can increase mitochondrial ROS (mtROS), disturb intracellular Ca²^+^ handling, and promote the release of mitochondrial components that participate in inflammatory signalling (32, 35). Lysosomal dysfunction can feed into the same network through altered Ca²^+^ flux and secondary mitochondrial damage (30, 36).

Pharmacological NSCLC models provide evidence that substantial oxidative and organelle stress can engage inflammasome-associated signalling (15). In A549 and H1299 cells, Polyphyllin VI increased ROS production together with NLRP3-associated signalling, caspase-1 activation, IL-1β/IL-18 release, GSDMD cleavage and pyroptotic cell death (37). ROS scavenging with N-acetyl-L-cysteine attenuated cell death and NLRP3-associated responses, supporting ROS generation as an upstream component of the PPVI-induced phenotype (37). These findings demonstrate that malignant epithelial cells can engage inflammasome-associated pathways under marked pharmacological stress, but they do not establish constitutive NLRP3 activity in untreated NSCLC (37).

A more mechanistically resolved example is provided by osimertinib-conditioned macrophages (30). Osimertinib induced lysosomal dysfunction and vacuolisation followed by lysosomal Ca²^+^ release into the cytosol (30). Functional experiments implicated TRPML1-dependent lysosomal Ca²^+^ signalling, and interference with this pathway reduced mitochondrial injury, ROS accumulation, NLRP3 activation and IL-1β release (30). This sequence provides direct evidence for a lysosome–Ca²^+^–mitochondria–NLRP3 axis in a treatment-conditioned NSCLC setting (30, 36).

Mechanistic studies outside NSCLC further strengthen the link between mitochondrial function and NLRP3 competence (35, 38). Billingham et al. showed that mitochondrial electron transport chain function is required to sustain efficient NLRP3 inflammasome activation, linking mitochondrial bioenergetics to inflammasome competence (38). These findings provide mechanistic plausibility for a mitochondrial contribution to treatment-associated NLRP3 activation, although direct validation in defined NSCLC cellular compartments remains necessary (35, 38). Taken together, current evidence supports an interconnected lysosome–Ca²^+^–mitochondria stress axis rather than entirely independent lysosomal and mitochondrial mechanisms (30, 35, 36). Importantly, oxidative stress and mitochondrial damage are not specific to NLRP3, and their mechanistic relevance is strongest when perturbation of the upstream stress response alters downstream inflammasome activity (8, 35).

### 3.2 Metabolic stress and DNA-sensing pathways

Metabolic perturbation can promote NLRP3 activation when it generates defined secondary stress signals, particularly mitochondrial dysfunction, ROS accumulation or cytosolic DNA sensing (11, 35). Changes in nucleotide metabolism, lipid handling and mitochondrial substrate utilisation can substantially alter redox balance and organelle integrity, but metabolic remodelling alone should not be interpreted as evidence of productive inflammasome activation (7, 35).

A direct NSCLC-related example is provided by a LUAD-associated macrophage model in which increased UPP1 disrupted nucleotide homeostasis, leading to mtROS accumulation and mtDNA leakage (39). These mitochondrial stress signals activated cGAS–STING signalling and subsequently engaged the NLRP3 inflammasome, resulting in IL-1β release (39). Functionally, this macrophage inflammatory programme promoted epithelial–mesenchymal transition, invasion and metastatic behaviour in LUAD cells, whereas interference with the pathway attenuated the tumour-promoting phenotype (39). This study therefore provides mechanistically informative, but incompletely resolved, evidence linking metabolic dysregulation and mitochondrial stress to a tumour-promoting macrophage NLRP3 programme in LUAD (35, 39).

The relationship between cGAS–STING and NLRP3 is nevertheless context dependent rather than obligatory (39, 40). Cytosolic DNA released after mitochondrial injury or treatment-associated tissue damage can activate cGAS–STING without necessarily engaging NLRP3 (35, 39). Following cryoablation, tumour-derived DNA activated macrophage cGAS–STING signalling and promoted CXCL10-dependent immune recruitment, but NLRP3 dependence was not demonstrated (41). This distinction is important because activation of an upstream danger-sensing pathway should not be taken as evidence of downstream inflammasome engagement in the absence of activation-specific readouts (8).

NSCLC-specific studies also define metabolic macrophage states that could provide a context in which inflammasome competence may vary, without directly demonstrating NLRP3 activation (42). In EGFR-driven LUAD, tumour-derived GM-CSF reprogrammed alveolar macrophage PPARγ-dependent lipid metabolism and cholesterol efflux, thereby supporting tumour-cell EGFR signalling and growth (42). Because NLRP3 expression, activation or pathway dependence was not directly assessed, this study is best interpreted as defining an NSCLC-specific metabolic context rather than as evidence of an active NLRP3 inflammasome (7, 42).

Mechanistic evidence from inflammatory macrophage systems further indicates that lipid metabolism can directly regulate NLRP3 (43). Leishman et al. showed that fatty-acid synthesis promotes NLRP3 inflammasome activation through FASN-dependent palmitoylation of NLRP3 (43). Together with the mitochondrial electron-transport findings described above, these data support the broader concept that metabolic state can regulate NLRP3 competence through both organelle-dependent and direct post-translational mechanisms (7, 35, 43). However, the relevance of these mechanisms to specific tumour-cell or macrophage populations in NSCLC remains to be established experimentally.

### 3.3 Therapy-induced stress as a major context for NLRP3 activation

Several of the best-characterised NLRP3-related responses in NSCLC arise after therapeutic or pharmacological exposure (30, 44). Treatment can simultaneously induce lysosomal injury, mitochondrial dysfunction, oxidative stress, Ca²^+^ imbalance, DNA damage and regulated cell death, thereby creating conditions in which multiple upstream stress pathways converge on inflammasome machinery (7, 32).

The available tumour-cell models illustrate this convergence. Polyphyllin VI and cucurbitacin B induce oxidative and mitochondrial stress together with inflammasome- and pyroptosis-associated responses in NSCLC cells (37, 45). These pharmacological models establish that sufficiently strong treatment-induced stress can drive malignant epithelial cells into inflammatory cell-death states, although the degree of NLRP3-specific functional dependence differs between studies (37, 45, 46).

Additional pharmacological evidence has been reported for rabeprazole (47). In lung-cancer models, rabeprazole suppressed tumour progression together with changes in NLRP3-, caspase-1- and pyroptosis-associated readouts (47). Because pathway-selective genetic or assembly-level validation was limited, these findings are more appropriately interpreted as drug-associated NLRP3-related inflammatory cell death rather than definitive evidence of NLRP3-specific dependence (8, 47).

Treatment-conditioned myeloid NLRP3 activation may arise through distinct proximal mechanisms (7, 30). In the osimertinib setting, lysosomal dysfunction and Ca²^+^ release were linked to secondary mitochondrial stress and macrophage NLRP3 activation (30). Mechanistic studies outside NSCLC-specific models provide additional support for this concept. Neuwirt et al. showed that several tyrosine kinase inhibitors can activate NLRP3 in primary myeloid cells through lysosomal damage, membrane destabilisation and subsequent lytic cell death (48). These observations reinforce the biological plausibility of therapy-conditioned myeloid NLRP3 activation, but such responses cannot be assumed to occur uniformly across TKIs, myeloid states or NSCLC treatment settings (7, 30, 48).

Therapy should therefore be regarded as an integrating biological context rather than as a single NLRP3 trigger (7, 46). Drug-induced activation demonstrates that the pathway can become functionally engaged under treatment pressure, but it does not establish an equivalent state in untreated disease (7, 30). Overall, current evidence supports a model in which treatment-related NLRP3 activation may emerge from convergent lysosomal, mitochondrial, metabolic and damage-sensing stress, with the initiating mechanism and biological consequence depending strongly on the affected cellular compartment (7, 30, 46). Representative studies and their corresponding levels of mechanistic support are summarised in **Table 2**.

**Table 2:** Examples illustrating application of the NLRP3 evidence framework in NSCLC.

Study/evidence component	Species and experimental context	Treatment/stimulus	Cellular source	Sample size/replication	Directly assessed readouts	Perturbation	Framework classification	Evidence source	Interpretation/principal limitation
Lasithiotaki et al., 2018 (31) – treatment-naïve human component	Human; treatment-naïve lung-cancer cohort with ex vivo BAL and peripheral-blood analyses	Ex vivo inflammatory stimulation	BAL alveolar macrophages; peripheral leukocytes	n = 31 participants: 15 NSCLC, 4 SCLC and 12 controls; qPCR performed in duplicate	NLRP3 expression; inducible IL-1β/IL-18 responses	No NLRP3-specific perturbation	A1/A4; B2	Direct NSCLC evidence	Demonstrates compartment-specific inflammasome-associated responsiveness in untreated disease. Cytokine responses do not establish NLRP3 as the upstream inflammasome sensor.
Yu et al., 2025 (30) – macrophage mechanistic component	Mouse BMDMs including gene-deficient macrophages; complementary human macrophage-related experiments	Osimertinib	Macrophages	3 independent experiments; quantified assays n = 3 or 4	ASC/NLRP3-related assembly; active CASP1; mature IL-1β; pyroptosis-associated responses	Nlrp3−/−, Pycard−/− and Casp1−/− BMDMs; Aim2−/−, Mevf−/− and Nlrc4−/− controls; CY-09/MCC950	A2/A3/A4; B1	Direct NSCLC evidence	NLRP3-specific loss of function together with independent Pycard/Casp1 dependence and competing-inflammasome controls satisfies the minimum B1 criteria in a treatment-conditioned macrophage setting.
Yu et al., 2025 (30) – murine tumour functional component	LLC-bearing WT and Nlrp3−/− mice	Osimertinib ± NLRP3 inhibition or recombinant IL-1β	Tumour-associated macrophage/tumour immune compartment	WT vs Nlrp3−/−: n = 5/group; osimertinib ± CY-09: n = 6/group; osimertinib ± rIL-1β: n = 4/group; tumour IL-1β assay: n = 4 biologically independent mice; *in vivo* data representative of 2 independent experiments	Tumour growth; tumour IL-1β; intratumoral macrophage CASP1 activation; T-cell remodelling	Nlrp3 deficiency; pharmacological NLRP3 inhibition; recombinant IL-1β	A3/A4; B2	Direct NSCLC evidence	NLRP3 perturbation supports *in vivo* pathway relevance, but independent ASC/PYCARD or CASP1 causal dependence was not demonstrated within this animal component; therefore B1 is not assigned.
Yu et al., 2025 (30) – human observational component	Human EGFR T790M-mutant NSCLC receiving second-line osimertinib	Osimertinib	Tumour-associated macrophage compartment	n = 47 patients; CD68+NLRP3+ high/low groups n = 24/23; CD68+IL-1β+ high/low groups n = 24/23	Macrophage NLRP3 and IL-1β expression	None	A1; B2	Direct NSCLC evidence	Supports treatment-associated clinical correlations but does not establish NLRP3 dependence or productive canonical inflammasome activation.
Feng et al., 2026 (39) – macrophage mechanistic component	Human THP-1 macrophage model and murine BMDMs	UPP1 overexpression/metabolic disturbance	Macrophages	n = 3 independent experiments for the NLRP3/CASP1/GSDMD/IL-1β mechanistic assays	ASC oligomerisation; NLRP3 upregulation; cleaved CASP1; GSDMD cleavage; LDH release; pyroptotic morphology; mature IL-1β secretion	shNLRP3; shGSDMD; CASP1 inhibitor VX-765; MitoTEMPO; cGAS inhibitor RU.521	A2/A3/A4/A5; B1	Direct NSCLC evidence	shNLRP3 suppressed IL-1β release and CASP1/GSDMD cleavage, with independent ASC-assembly and CASP1-pathway evidence. This component therefore meets the predefined B1 minimum criteria.
Teng et al., 2020 (37) – *in vitro* tumour-cell component	Human A549 and H1299 NSCLC cells	Polyphyllin VI	Tumour cells	≥3 independent biological experiments	Cleaved CASP1; mature/cleaved IL-1β and IL-18; cytokine release; GSDMD cleavage; pyroptotic morphology/lysis	CASP1 inhibition and downstream pyroptosis-pathway manipulation	A3/A4/A5; B2	Direct NSCLC evidence	Demonstrates treatment-induced canonical-inflammasome/pyroptosis-associated responses, but NLRP3-specific causal dependence was not demonstrated.
Teng et al., 2020 (37) – *in vivo* xenograft component	A549 xenograft mouse model	Polyphyllin VI	Tumour xenograft/malignant epithelial compartment	n = 8 mice/group; five treatment groups	Tumour inhibition; NLRP3; cleaved CASP1; cleaved IL-1β; cleaved GSDMD	Pharmacological treatment; no NLRP3-specific genetic perturbation	A3/A4/A5; B2	Direct NSCLC evidence	Supports *in vivo* inflammasome/pyroptosis engagement but does not independently establish NLRP3-specific causal dependence.
Yuan et al., 2021 (45) – *in vitro* tumour-cell component	Human A549 NSCLC cells	Cucurbitacin B	Tumour cells	n ≥ 3 independent experiments where explicitly specified; otherwise biological replication not reported	NLRP3–NEK7 interaction; ASC specks; cleaved CASP1; mature IL-1β; GSDMD-N; pyroptotic morphology	TLR4 silencing; pharmacological pathway manipulation	A2/A3/A4/A5; B2	Direct NSCLC evidence	Multiple canonical inflammasome and pyroptotic events were demonstrated, but the principal genetic perturbation targeted TLR4 rather than NLRP3.
Yuan et al., 2021 (45) – *in vivo* syngeneic component	LLC-bearing C57BL/6J mice	Cucurbitacin B	Tumour/tumour microenvironment	n = 8 mice/group at treatment allocation	Tumour response; NLRP3/GSDMD-associated pyroptotic phenotype	Pharmacological treatment; TLR4-centred pathway manipulation	A5; B2	Direct NSCLC evidence	Supports an *in vivo* TLR4/NLRP3-associated pyroptotic phenotype but does not provide NLRP3-specific genetic causality.
Ye et al., 2025 (51) – transcriptional-regulatory component	Human NSCLC cells	NET exposure/MIR503HG–C/EBPβ regulation	Tumour cells	NLRP3 transcriptional/regulatory assays: n = 3 independent experiments; migration assays: n = 3; invasion assays: n = 5 where specified	NLRP3 mRNA/protein; C/EBPβ binding to the NLRP3 promoter	MIR503HG and C/EBPβ manipulation	A1; B2	Direct NSCLC evidence	Supports regulation of NLRP3 expression associated with metastatic behaviour but does not establish productive canonical inflammasome activation.

A- and B-axis categories are assigned to individual experimental evidence components rather than entire publications. Human observational, *in vitro* mechanistic and *in vivo* animal components are separated when they support distinct conclusions. Sample size and biological replication are reported at the corresponding experimental-component level whenever explicitly stated in the primary article, Methods, **Supplementary Material** or figure legends. Where biological replication or group size is not reported, this is stated explicitly as “not reported.” Technical replicates are distinguished from independent biological replicates. B1 requires both NLRP3-specific causal perturbation and independent evidence supporting canonical inflammasome involvement; NLRP3 perturbation alone is insufficient for B1. B2 includes NLRP3-associated findings without demonstrated NLRP3 dependence and NLRP3-dependent phenotypes for which canonical inflammasome involvement remains unresolved. B3 requires NLRP3 dependence together with evidence that canonical NLRP3–ASC/PYCARD–CASP1 signalling is dispensable.

## 4 Cellular sources and context-dependent NLRP3-associated biology in NSCLC

The biological consequences of NLRP3-associated signalling in NSCLC vary according to the cellular compartment and experimental context in which NLRP3 activation is inferred, observed or experimentally induced (49). Available mechanistic and functional studies have primarily examined malignant epithelial cells and macrophages, but the level of NLRP3-specific mechanistic support differs between these settings (49). In tumour cells, treatment-induced inflammatory cell death represents a recurrent experimentally observed phenotype, although the contribution of NLRP3-specific inflammasome signalling has not been uniformly established across studies (44). By contrast, macrophage-associated NLRP3 signalling has been linked to both antitumour immune remodelling and tumour-promoting inflammatory communication, reflecting the context-dependent functions of NLRP3 within the tumour microenvironment (49). Pathway-specific mechanistic support has been demonstrated only in selected treatment-conditioned models (30). Neutrophils and other immune or stromal populations contribute to the broader inflammatory architecture of NSCLC, although direct evidence establishing cell-intrinsic NLRP3-dependent function in these compartments remains limited (14). From the perspective of mechanistic attribution, studies classified as B2 should not be interpreted as demonstrating a defined NLRP3-dependent mechanism, but rather as NLRP3-associated observations requiring further mechanistic validation (9). This category includes findings with incomplete pathway attribution, such as expression-based associations, pharmacological effects without adequate target validation or orthogonal confirmation, or phenotypes for which canonical inflammasome involvement remains unresolved (11, 50).

### 4.1 Malignant epithelial cells: inflammatory cell death and tumour-state regulation

In Polyphyllin VI- and cucurbitacin B-treated models, the principal cellular outcome is inflammatory tumour-cell death characterised by caspase-1- and GSDMD-associated pyroptotic execution (37, 45). However, the depth of pathway-specific validation differs between studies, and these models more clearly establish inducible inflammatory death under pharmacological stress than constitutive NLRP3 activation in untreated human NSCLC (11).

Rabeprazole provides an additional example in which NLRP3-, caspase-1- and pyroptosis-associated changes accompanied tumour suppression, although NLRP3-specific dependence remains incompletely established (9, 47). Collectively, these pharmacological models support the capacity of NSCLC epithelial cells to enter inflammasome-associated death programmes under strong experimental stress, while leaving uncertain how frequently such states occur spontaneously in untreated tumours (11, 49).

NLRP3-associated tumour-cell biology is not restricted to pyroptotic execution (49, 51). NET exposure has been linked to a MIR503HG–C/EBPβ–NLRP3 regulatory axis in NSCLC cells (51). Mechanistically, MIR503HG promotes the ubiquitination and degradation of C/EBPβ, whereas NET-associated suppression of MIR503HG increases C/EBPβ availability and enhances C/EBPβ-dependent NLRP3 transcription (51). This regulatory shift was associated with increased migration, invasion and metastatic behaviour of NSCLC cells (51). These findings therefore support an NLRP3-associated transcriptional programme linked to tumour-promoting phenotypes, rather than establishing productive canonical inflammasome activation (11, 51).

Importantly, increased NLRP3 transcription in this context should not automatically be equated with productive canonical inflammasome activation (9). The tumour-cell literature therefore supports at least two separable patterns: treatment-induced inflammasome/pyroptosis-associated cell death and NLRP3-associated transcriptional regulation linked to malignant behaviour (45, 51). Within the evidence framework used here, both patterns remain distinct from NLRP3-specific mechanistic dependence unless supported by pathway-specific perturbation. Whether either pattern is broadly present in untreated human NSCLC remains uncertain (9).

### 4.2 Tumour-associated macrophages: context-dependent effects on tumour control and progression

Macrophages represent a cellular setting in which NLRP3-specific mechanistic support has been demonstrated in selected NSCLC-related experimental contexts (30). Importantly, macrophage NLRP3 signalling does not have a uniform biological consequence (49). Treatment-naïve human evidence also indicates compartment-specific inflammasome-associated responses. In patients with primary lung cancer, including 15 with NSCLC, Lasithiotaki et al. found increased basal IL-1β/IL-18 secretion and inducible IL-1β responses in peripheral blood leukocytes, whereas BAL-derived alveolar macrophages showed impaired LPS/ATP-induced IL-1β/IL-18 responses and reduced NLRP3 mRNA expression. These findings demonstrate divergent peripheral and lung-resident response profiles but do not establish NLRP3-specific causal dependence (31).

In an osimertinib-conditioned experimental setting, canonical macrophage NLRP3 signalling contributed to IL-1β-associated immune remodelling and supported therapeutic efficacy (30). NLRP3-, ASC/PYCARD- and CASP1-directed perturbation, together with pharmacological NLRP3 inhibition and competing-inflammasome controls, provided mechanistic support for canonical NLRP3 signalling in this treatment-conditioned setting (30). Pharmacological inhibition of NLRP3 reduced the antitumour effect, whereas exogenous IL-1β enhanced treatment responsiveness (30). Associations between macrophage NLRP3/IL-1β expression and outcomes in patients receiving EGFR-targeted therapy provide additional clinical support, although these patient-level observations remain correlative (9, 30). Accordingly, the mechanistic experimental component and the correlative human component should be interpreted separately (9).

A contrasting macrophage programme has been reported in LUAD in association with UPP1 dysregulation (39, 49). In this setting, NLRP3-related inflammatory signalling and IL-1β release formed part of a macrophage-to-tumour inflammatory circuit that promoted epithelial–mesenchymal transition, invasion and metastatic behaviour (39). Interference with components of this pathway attenuated the tumour-promoting phenotype (39). However, NLRP3-specific dependence was not established to the same extent as in the osimertinib-conditioned model; this evidence is therefore more appropriately interpreted as NLRP3-associated rather than as equivalent pathway-specific mechanistic evidence (11).

These findings indicate that macrophage NLRP3-associated signalling can occur in biologically divergent contexts associated with either tumour control or tumour progression (30, 39, 49). The difference is unlikely to be explained by macrophage identity alone; rather, the initiating stimulus, metabolic state, treatment context, downstream inflammatory output and identity of neighbouring responding cells are likely to shape the biological consequence (52, 53).

Single-cell studies further emphasise macrophage heterogeneity in NSCLC (54). Multiple transcriptional macrophage states, including SPP1^+^ and other tissue- or inflammation-associated populations, have been identified across NSCLC and broader cancer single-cell datasets (55). These populations provide candidate cellular contexts in which inflammasome competence may differ, but transcriptional state alone is insufficient to infer inflammasome-associated responsiveness (11). Accordingly, these transcript-defined macrophage states are treated as contextual or association-level evidence rather than as evidence of spatially localised NLRP3-dependent activity. Direct NLRP3-specific functions therefore cannot yet be assigned reliably to most individual macrophage subsets (11, 55).

Metabolically reprogrammed macrophage states should not be equated with functionally NLRP3-active states in the absence of activation-specific and pathway-specific evidence (11). Likewise, spatial localisation or proximity of macrophage subsets to tumour or stromal compartments provides contextual information on cellular organisation but does not establish NLRP3-dependent cellular interactions (56).

### 4.3 Neutrophils and other microenvironmental compartments: predominantly indirect roles

Direct evidence for cell-intrinsic NLRP3 activity in neutrophils and other non-macrophage microenvironmental compartments remains limited in NSCLC (57). In NSCLC, neutrophils appear to be relevant primarily through their participation in multicellular inflammatory circuits (57). NETs can alter NLRP3-associated transcriptional states in neighbouring NSCLC cells (51), but this does not establish neutrophil-intrinsic NLRP3 activation (11). Inflammatory mediators generated by other myeloid populations may likewise influence neutrophil recruitment and activation (57), yet direct demonstration of a dominant neutrophil-intrinsic NLRP3 programme in NSCLC remains limited (58). These observations therefore support a contextual role for neutrophils rather than direct evidence of neutrophil-intrinsic NLRP3 dependence (11).

Dendritic cells and lymphoid populations are central to antigen presentation, T-cell priming and the organisation of adaptive immune responses, including tertiary lymphoid structures (59). Inflammasome-associated cytokines may influence these processes indirectly, but available NSCLC-specific evidence does not yet establish a major dendritic-cell- or lymphocyte-intrinsic NLRP3 programme (11). Accordingly, lymphoid and TLS-associated regions should be considered candidate microenvironmental niches rather than NLRP3-defined spatial states (56).

Fibroblasts and endothelial cells similarly shape extracellular-matrix architecture, vascular access, cytokine gradients and immune-cell localization (60). These compartments may therefore influence where NLRP3-associated tumour or myeloid states arise and which neighbouring cells are exposed to inflammatory outputs (55, 60). However, current evidence does not establish fibroblasts or endothelial cells as major sources of functionally active NLRP3 inflammasomes in NSCLC (11). Stromal/CAF-rich and endothelial regions should therefore be interpreted as candidate contextual niches unless inflammasome-associated responsiveness is directly demonstrated (11, 60).

For these less well-defined compartments, their present relevance lies primarily in their capacity to organise or respond to broader inflammatory networks in which NLRP3-associated signals may occur, rather than in firmly established cell-intrinsic inflammasome activity (11, 52). Spatial proximity, transcript abundance and inferred communication should not be interpreted as evidence of inflammasome assembly, CASP1 activation or NLRP3-dependent intercellular signalling (11, 56).

Finally, NLRP3-dependent effects should be distinguished from canonical inflammasome-dependent effects (9, 11). Inflammasome-independent functions of NLRP3 have been reported in several non-NSCLC systems, including transcriptional regulation and modulation of TGF-β-associated profibrotic signalling (20, 21). No NSCLC evidence component included in the present framework currently provides sufficient pathway-separation evidence to establish a B3 inflammasome-independent NLRP3 function (11). Accordingly, these mechanisms should be regarded as mechanistic hypotheses for future testing rather than as established tumour-cell or stromal functions in this disease (11).

## 5 Spatial context for interpreting NLRP3-associated biology in NSCLC

The spatial organisation of malignant, immune and stromal populations provides an important context for interpreting inflammatory states in NSCLC (22, 61). Spatial transcriptomics, multiplex imaging and integrated single-cell approaches have revealed substantial regional heterogeneity in immune-cell composition, stromal architecture and tumour–immune organisation (22, 61). However, these observations should be distinguished from evidence of functional NLRP3 activity (12). The spatial studies included in the present evidence map do not, by themselves, demonstrate functional NLRP3 activation, which requires evidence beyond transcriptional abundance or spatial colocalisation, such as inflammasome assembly, caspase-1 activation, maturation of IL-1β/IL-18 or downstream gasdermin activation (7). They also do not establish cellular directionality or NLRP3-dependent intercellular interactions. Accordingly, spatial evidence is considered here in three categories: direct functional NLRP3 evidence, NLRP3-related transcriptional evidence, and candidate cellular or tissue niches identified without direct NLRP3 interrogation.

### 5.1 Direct functional NLRP3 evidence in spatially resolved NSCLC

Among the spatially resolved NSCLC studies included in the present evidence map, none combines activation-specific measurements with NLRP3-specific perturbation sufficient to establish a functionally NLRP3-dependent spatial state (7). Consequently, spatially defined cell populations or neighbourhoods should not currently be designated as NLRP3-active niches on the basis of spatial data alone (7, 62). Mechanistic findings obtained in non-spatial experimental systems remain relevant to pathway biology, but they do not establish where an equivalent NLRP3-dependent state occurs within human NSCLC tissue (62). Demonstrating such a spatial state would require cell-resolved localisation together with activation-specific readouts and pathway-specific perturbation (7, 62).

### 5.2 NLRP3-related transcriptional evidence

Among the spatially resolved studies included in the present evidence map, we did not identify a study that directly reported a sufficiently specific NLRP3-related transcript or transcriptional programme to justify classification as NLRP3-related transcriptional evidence. Studies were not assigned to this category solely because their datasets could in principle be re-analysed for NLRP3; classification requires that NLRP3-related expression or priming findings are explicitly reported in the primary study.

This distinction is important because NLRP3 or IL1B transcript abundance reflects pathway expression or inflammatory priming rather than inflammasome assembly or productive downstream activation (11, 32). Transcript-based pathway scores and inferred inflammatory states therefore remain contextual evidence unless supported by activation-specific measurements (7, 12). Such transcriptional observations may identify candidate cell populations or tissue regions for subsequent functional investigation, but should not be interpreted as evidence of functional NLRP3 inflammasome activity.

### 5.3 Candidate cellular and spatial niches without direct NLRP3 interrogation

Spatially resolved NSCLC studies considered here define cellular states, neighbourhoods and tissue architectures that provide relevant microenvironmental context but do not directly establish NLRP3 activity.

De Zuani et al. integrated single-cell RNA sequencing with spatial transcriptomics in treatment-naïve NSCLC and adjacent non-malignant tissues, characterising tumour and immune-cell states and macrophage-associated transcriptional programmes (22). The primary analysis did not report a specific NLRP3-related transcriptional result; accordingly, this study is classified here as candidate cellular/spatial context without direct NLRP3 interrogation rather than as NLRP3-related transcriptional evidence. This treatment is consistent with the evidence framework, in which spatial or transcriptional context alone is not considered evidence of functional NLRP3 pathway activity.

Sorin et al. characterised spatial immune landscapes in LUAD and identified heterogeneous distributions of immune populations across histological contexts (61). Enfield et al. identified distinct myeloid and lymphoid spatial architectures in treatment-naïve early-stage lung cancers (63), while Wang et al. reported histology-associated tumour-cell states and macrophage distributions using spatial transcriptomic and multiplex approaches (64). These studies establish spatial heterogeneity within the NSCLC microenvironment but do not establish functional NLRP3 activity in the identified cell populations. Spatial transcriptomic measurements define tissue architecture and cell-state relationships but do not, by themselves, demonstrate inflammasome activation or pathway dependence (10, 62).

Additional single-cell and spatial studies have characterised stromal–immune configurations potentially relevant to inflammatory signalling. Chen et al. reported POSTN^+^ cancer-associated fibroblast states associated with immune suppression and tumour progression in NSCLC (65), while Yan et al. identified multi-omic features associated with resistance to immuno-chemotherapy (66). Such observations provide candidate anatomical or cellular contexts for subsequent investigation but do not establish NLRP3-dependent signalling or the directionality of inflammatory interactions.

Spatial immune niches and organised immune structures provide additional contextual information. Sandström Gerdtsson et al. characterised spatial immune-infiltration niches in NSCLC (67), while Chen et al. identified spatially organised stem–immunity hubs associated with immunotherapy response in lung cancer (68). Tertiary lymphoid structures represent another component of tumour immune organisation (69). Their presence or spatial association with other cell populations should not, by itself, be interpreted as evidence of NLRP3 inflammasome activity.

Treatment-associated spatial analyses provide a further layer of clinically annotated context. Aung et al. identified spatial signatures associated with immunotherapy outcomes in NSCLC (70), whereas Zhao et al. characterised spatial immune-microenvironment features in KRAS-mutant NSCLC in relation to immunotherapy outcome (71). These studies did not directly establish NLRP3 activation or NLRP3-specific pathway dependence and are therefore considered contextual rather than mechanistic NLRP3 evidence.

Taken together, current single-cell and spatial studies define cellular states and spatial microenvironmental contexts relevant to inflammatory biology rather than demonstrating that functional NLRP3 activity is spatially organised in NSCLC. Designation of a spatially localised NLRP3-active state would require activation-specific evidence, such as inflammasome assembly or downstream caspase-1/IL-1β processing, together with NLRP3-specific causal support (7, 18). Subsequent spatial analysis could then determine whether such a validated state occupies reproducible cellular niches or changes with treatment (10, 62).

## 6 Therapeutic perspectives of NLRP3-associated inflammation in NSCLC

The therapeutic relevance of NLRP3-associated inflammation in NSCLC depends on whether functionally demonstrated pathway activity can be linked to a clinically relevant disease state amenable to therapeutic modulation (7, 72). NLRP3 itself, IL-1 signalling and pyroptotic execution can each, in principle, be modulated at different levels, but they are not equivalent therapeutic targets (7, 23). Recent structural and pharmacological studies have expanded the range of experimentally tractable points within NLRP3 signalling (7, 73). Most of this work, however, has arisen from inflammatory disease settings rather than oncology, and clinical evidence for direct NLRP3 inhibition in NSCLC is currently lacking (23, 72).

The immediate translational question is therefore not pathway druggability alone, but whether functionally demonstrated NLRP3 activity contributes causally to a clinically relevant phenotype that would justify intervention. This need for biological selection is further supported by treatment-naïve human evidence showing distinct inflammasome-associated responses in peripheral blood cells and BAL-derived alveolar macrophages (31).

### 6.1 Direct NLRP3 inhibition

Small-molecule NLRP3 inhibitors remain valuable for mechanistic interrogation and have helped define conformational requirements for inflammasome activation and pharmacological suppression (7, 73). Structural and functional analyses of NLRP3 have shown that alterations in ATP-dependent regulation, conformational stability and oligomerisation can influence pathway activity and inhibitor sensitivity (73). These findings refine the mechanistic basis of NLRP3 inhibition, but they should not be interpreted as evidence of therapeutic efficacy in NSCLC (23, 73).

Current NSCLC studies indicate that inflammatory myeloid states are highly context-dependent, while NLRP3 biology cannot be inferred from pathway abundance alone because inflammasome activation depends on regulatory context, assembly processes and downstream effector engagement (23, 36, 61). Direct inhibition would therefore be difficult to justify as an unselected anti-inflammatory strategy (7).

Accordingly, direct NLRP3 inhibition is better regarded as a context-dependent therapeutic hypothesis than as a strategy applicable to NSCLC in general (7, 72). Clinical development would be more convincingly supported by evidence that the targeted NLRP3 state is functionally engaged and biologically relevant in the treatment setting under investigation (7). Importantly, clinical trials of downstream IL-1β blockade do not provide direct clinical evidence for or against NLRP3 inhibition (23).

### 6.2 IL-1β blockade: lessons from clinical trials

IL-1β blockade provides the principal clinical experience with intervention at a downstream inflammasome-associated level, but it should be distinguished from direct NLRP3 inhibition (23, 74). Canakinumab is an IL-1β-neutralising monoclonal antibody and does not identify or selectively inhibit the upstream inflammasome responsible for IL-1β production (23, 74). Consequently, clinical responses to canakinumab cannot establish NLRP3 activation or NLRP3 dependence (23, 24). The major CANTOS and CANOPY trials are summarised quantitatively in **Supplementary Table S4**, including trial design, population, intervention, sample size, prespecified primary endpoint, effect estimates with 95% confidence intervals, statistical status, registration and principal safety findings (25, 74).

The initial lung-cancer signal arose from an exploratory cancer analysis of CANTOS, a randomised cardiovascular outcomes trial involving 10, 061 patients with previous myocardial infarction and elevated high-sensitivity C-reactive protein rather than patients undergoing treatment for established lung cancer (24, 74). The exploratory lung-cancer analysis of the CANTOS randomised trial reported lower incident lung-cancer rates at selected canakinumab doses, including an HR of 0.33 (95% CI 0.18–0.59) with the 300-mg dose, together with lower lung-cancer mortality at that dose (HR 0.23, 95% CI 0.10–0.54) (24). These findings were exploratory and hypothesis-generating; fatal infection or sepsis was more frequent with canakinumab in the parent CANTOS trial (74).

Prospective NSCLC trials did not demonstrate benefit on their prespecified primary efficacy endpoints. In CANOPY-1, 643 patients with previously untreated advanced NSCLC were randomised to canakinumab or placebo in combination with pembrolizumab and platinum-based chemotherapy (25). PFS was similar between groups (HR 0.85, 95% CI 0.67–1.09; P = .102), as was OS (HR 0.87, 95% CI 0.70–1.10; P = .123), with neither coprimary endpoint meeting its prespecified statistical criterion (25). CANOPY-A likewise did not meet its primary DFS endpoint after complete resection and adjuvant cisplatin-based chemotherapy (HR 0.94, 95% CI 0.78–1.14; one-sided P = .258), and OS was not formally tested because DFS was not statistically significant (26). CANOPY-2 did not meet its primary OS endpoint in previously treated advanced NSCLC (HR 1.06, 95% CI 0.76–1.48; one-sided P = .633) (27).

CANOPY-N provides a smaller neoadjuvant example in resectable NSCLC. MPR was 2.9% (95% CI 0.07–14.92) with canakinumab alone and 17.1% (95% CI 6.56–33.65) with canakinumab plus pembrolizumab (28). Neither canakinumab-containing arm met the prespecified proof-of-efficacy criteria, while the small treatment groups and wide confidence intervals around the observed MPR rates limit the precision of the efficacy estimates (28).

Taken together, these trials do not demonstrate clinical benefit from canakinumab-based IL-1β blockade in the NSCLC populations evaluated (25–28). They should not, however, be interpreted as negative clinical tests of direct NLRP3 inhibition (23, 25). Neutralising downstream IL-1β does not, by itself, identify the responsible upstream inflammasome sensor or establish that a tumour- or immune-cell phenotype is NLRP3-dependent (23, 24). Exploratory subgroup and biomarker observations should therefore remain hypothesis-generating unless prospectively validated (27, 74). Clinical evaluation of direct NLRP3 inhibition would require evidence of target engagement together with a biologically defined NLRP3-dependent disease state (7, 23).

### 6.3 Pyroptotic execution as a distinct therapeutic problem

Therapeutic induction of inflammatory tumour-cell death is conceptually adjacent to, but distinct from, direct NLRP3 targeting (19, 46). Pharmacological models show that NSCLC cells can enter gasdermin-associated death programmes under selected treatment conditions (75), while broader cancer research has generated interest in manipulating immunogenic cell stress and death to alter treatment responses (46). The clinical value of deliberately inducing such death programmes in NSCLC remains to be established, particularly given the marked heterogeneity of the lung tumour immune microenvironment (46, 63).

Recent work on NINJ1 is relevant here as mechanistic extrapolation rather than direct NSCLC evidence (76). Studies outside lung cancer have established terminal plasma-membrane rupture as a regulated event downstream of earlier membrane-permeabilisation steps (76). Structural analyses showed that NINJ1 polymerises into membrane-disruptive assemblies during lytic cell death (76), while antibody-mediated inhibition of NINJ1 reduced plasma-membrane rupture and inflammatory tissue injury in experimental models (77).

These findings mainly refine how pyroptotic phenotypes should be interpreted experimentally. Gasdermin activation, membrane permeabilisation and terminal rupture are related but non-identical events (19, 78). None of these downstream events, by itself, identifies the initiating upstream inflammasome (8, 23). Their relevance to NLRP3-directed therapy in NSCLC therefore requires upstream pathway validation (7, 23). Whether selective modulation of terminal membrane rupture itself can provide therapeutic benefit in lung cancer has not yet been established (19, 79).

### 6.4 From pathway activity to patient selection

The clinical value of an NLRP3-associated biomarker is unlikely to be captured by bulk pathway abundance alone (7, 23). More informative approaches would need to resolve the cellular compartment carrying the signal, evidence of functional pathway engagement and the treatment stage at which the state is detected (7, 66).

Spatial and multiplex technologies may support this level of resolution by linking protein readouts to cell identity while retaining tissue architecture (80). Their principal value in this setting is to assign candidate signals to defined cellular populations (81); spatial association, by itself, does not establish NLRP3 function; functional interpretation would still require appropriate pathway-specific evidence (7, 80).

Timing may be equally important. Where NLRP3-associated activity is treatment dependent, paired baseline and on-treatment specimens may help distinguish pre-existing pathway activity from a state that emerges or changes during therapy (7, 66). Longitudinal sampling could then determine whether such changes track with treatment response, residual disease or progression, without implying that temporal association itself establishes causality (66).

At present, these considerations favour evaluating NLRP3-directed strategies in biologically defined, pathway-validated NSCLC settings rather than assuming applicability across unselected disease (7, 63). Such selection may also reduce the risk of suppressing inflammasome-associated activity that could contribute to beneficial antitumour responses in specific treatment contexts (7, 46).

## 7 Outstanding questions and methodological priorities

The principal limitation of the current NSCLC literature is the small number of studies that connect a cell-resolved NLRP3 state to pathway dependence and then examine its relevance in human disease (15, 30). Future work would therefore benefit more from resolving these links than from expanding expression-based associations alone (15).

### 7.1 From association to pathway dependence

Mechanistic studies should be organised around the phenotype under investigation rather than around the presence of an NLRP3-related marker (8, 82). Once a candidate cellular source has been identified, perturbation of NLRP3 can be compared with disruption of canonical inflammasome components to determine which part of the pathway is required (8, 83). Rescue and pathway-separation experiments are particularly informative when an NLRP3-dependent phenotype appears to persist independently of canonical downstream outputs (84).

Such designs would address two recurrent sources of uncertainty: attribution of an inflammasome-associated phenotype to NLRP3 without sensor-specific evidence, and attribution of every NLRP3-dependent phenotype to canonical inflammasome signalling (8, 84). The depth of validation required will depend on the biological claim, but mechanistic conclusions should remain proportionate to the perturbation actually performed.

This approach extends the interpretative framework established earlier in the review without repeating it: the relevant question is no longer whether an NLRP3-associated signal is present, but which molecular component is necessary for the phenotype being studied.

### 7.2 Human relevance and model selection

Experimental models should be matched to the cellular process under investigation (85, 86). Established NSCLC cell lines permit controlled perturbation of tumour-cell pathways, whereas macrophage-centred questions require systems that preserve relevant myeloid biology and multicellular interaction (85). Patient-derived tumour cultures, organoids, tumour slices and tumour–immune co-cultures therefore provide complementary rather than interchangeable experimental settings (86, 87).

Species differences add a further constraint. NLRP11 contributes to NLRP3 inflammasome assembly in human macrophages but is not expressed in mice, illustrating that regulatory mechanisms identified in one species may not be conserved in another (88). Murine models remain valuable for studying integrated tumour–immune responses, but mechanistic findings intended to inform human treatment should, where feasible, be examined in an appropriate human system (85, 88).

The aim is therefore not to replace animal models with a single human platform, but to combine models in which cell-intrinsic signalling, multicellular interaction and organism-level treatment effects can each be tested at the appropriate level.

### 7.3 Functional validation across treatment states

Cross-sectional profiling can identify candidate NLRP3-associated states, but it cannot establish whether they precede treatment, emerge during therapy or become enriched during progression (70, 89). Longitudinal clinical sampling is therefore particularly relevant to a pathway for which some of the clearest NSCLC evidence is treatment conditioned (30).

A useful strategy would combine discovery in spatial or single-cell datasets with independent functional validation, followed by assessment in appropriately timed clinical specimens (86, 89). Spatial profiling can nominate candidate cellular populations or neighbourhoods (89), whereas activation-related protein measurements and ex vivo perturbation can test whether these candidates correspond to a functionally engaged pathway (80, 86).

Recent spatial outcome studies in NSCLC provide a methodological precedent for linking multicellular architecture with treatment response, although they do not establish NLRP3 involvement (70). Their relevance here lies in demonstrating how a pathway state, once functionally validated, could subsequently be tested for reproducible spatial and treatment-associated organisation (70).

### 7.4 Translational testing

Clinical association becomes more informative once cellular identity and pathway dependence have been established (86, 90). Candidate biomarkers would be more convincing if supported by reproducible localisation, evidence of functional engagement and an association with a predefined clinical endpoint after consideration of established clinical and molecular predictors (90).

Prospective cohorts are particularly useful for testing predefined hypotheses, although carefully annotated longitudinal retrospective material may also be informative (90). Internal validation and, where feasible, independent validation would reduce the likelihood that an NLRP3-associated signature simply reflects cohort-specific inflammatory composition rather than a reproducible biological state (90).

Therapeutic studies would additionally benefit from pharmacodynamic measurements showing whether the intended pathway has actually been modulated (7, 23). Without such evidence, a negative result could reflect inadequate target engagement, inappropriate patient selection or absence of therapeutic dependence, whereas a positive result might remain difficult to attribute specifically to NLRP3 (7).

Together, these considerations suggest that cellular origin and pathway dependence should be established before biomarker or therapeutic significance is assigned, with spatial and longitudinal analyses used to determine where and when the validated state occurs (86, 89).

## 8 Conclusion

Current evidence does not support NLRP3 as a uniform therapeutic determinant across NSCLC (7, 91). Rather, the available literature indicates that NLRP3-related findings represent heterogeneous biological states that differ in measured pathway events, mechanistic attribution, cellular compartment, treatment status and experimental context (30, 31, 91). A more informative distinction is therefore between NLRP3-associated observations and experimental states in which NLRP3-specific pathway dependence has been demonstrated sufficiently to permit mechanistic interpretation (7).

This distinction is also important therapeutically. Direct NLRP3 inhibition, IL-1β neutralisation and modulation of pyroptotic execution intervene at different levels of inflammatory signalling, and evidence for one strategy cannot be used to infer the efficacy or biological relevance of another (7). The CANOPY trials illustrate this distinction: the absence of clinical benefit from IL-1β neutralisation in the populations studied does not resolve whether a biologically selected NSCLC subgroup with demonstrated NLRP3 dependence might benefit from direct NLRP3 inhibition (7, 25, 26).

The next stage of the field is therefore to determine whether such NLRP3-dependent states can be reproducibly identified in human NSCLC, whether they are restricted to specific cellular or treatment contexts, and whether they provide information beyond established cellular, molecular and treatment-related features (30, 63). Until these questions are resolved, NLRP3 is better regarded as a context-dependent mechanistic pathway requiring further validation for biomarker or therapeutic use, rather than as a general biomarker or therapeutic target in NSCLC (7, 30).
