# Supplementary material for: NLRP3 signalling in NSCLC: distinguishing expression, inflammasome activation and context-dependent function
Source: Front Immunol. 2026 Oct 20;17:1983973. doi: 10.3389/fimmu.2026.1983973 (PMC13729371; doi:10.3389/fimmu.2026.1983973)
Supplement: Supplementary file 1 [file Supplementaryfile1.docx]

Supplementary Material

# 1 Supplementary Tables

## 1.1 Supplementary Table S1

## Table S1. Structured evidence map of primary studies retained within the predefined evidence framework for NLRP3-associated biology and relevant spatial/contextual evidence in NSCLC and lung cancer.

| **Study** | **Evidence component** | **Species / model / population** | **Sample size / experimental replication** | **Treatment / stimulus** | **Cellular source** | **Key readouts** | **Perturbation** | **Principal finding** | **A category** | **B category** | **Evidence source** | **Principal limitation** |
| --- | --- | --- | --- | --- | --- | --- | --- | --- | --- | --- | --- | --- |
| **Lasithiotaki et al., 2018 (31)** | Treatment-naïve human ex vivo inflammatory-response component | Human lung-cancer cohort | n = 31 participants: 15 NSCLC, 4 SCLC and 12 controls; qPCR performed in duplicate | Ex vivo inflammatory stimulation | BAL alveolar macrophages; peripheral leukocytes | NLRP3 expression; inducible IL-1β/IL-18 secretion | No NLRP3-specific perturbation | Inducible cytokine responses differed between BAL macrophages and peripheral leukocytes, indicating compartment-specific inflammatory responsiveness | A1/A4 | B2 | Direct NSCLC evidence | NLRP3 expression and cytokine output do not identify NLRP3 as the causal upstream inflammasome sensor |
| **Yu et al., 2025 (30)** | Macrophage mechanistic component | WT and gene-deficient murine BMDMs; complementary human macrophage-related experiments | 3 independent experiments; quantified assays: n = 3 or 4 | Osimertinib | Macrophages | Active CASP1; mature IL-1β; inflammasome-assembly-associated readouts | *Nlrp3*−/−, *Pycard*−/− and *Casp1*−/− BMDMs; *Aim2*−/−, *Mefv*−/− and *Nlrc4*−/− controls; CY-09 and MCC950 | Demonstrates canonical NLRP3-dependent inflammasome signalling following osimertinib exposure | A2/A3/A4 | B1 | Direct NSCLC evidence | B1 is supported by NLRP3-specific loss of function together with independent PYCARD/CASP1 dependence and competing-inflammasome controls; the model is treatment-conditioned |
| **Yu et al., 2025 (30)** | Murine tumour functional component | LLC-bearing WT and *Nlrp3*−/− mice | WT versus *Nlrp3*−/−: n = 5/group; osimertinib ± CY-09: n = 6/group; osimertinib ± rIL-1β: n = 4/group; tumour IL-1β assay: n = 4 biologically independent mice; representative of 2 independent experiments | Osimertinib ± pathway manipulation | Tumour-associated macrophages and broader tumour immune compartment | Tumour growth; intratumoural macrophage CASP1 activation; IL-1β; T-cell remodelling | *Nlrp3* deficiency; pharmacological NLRP3 inhibition; recombinant IL-1β | Supports an in vivo contribution of the NLRP3–IL-1β axis to osimertinib-associated tumour control | A3/A4 | B2 | Direct NSCLC evidence | NLRP3 perturbation was demonstrated, but independent PYCARD or CASP1 causal perturbation was not performed within this animal component; therefore, B1 is not assigned |
| **Yu et al., 2025 (30)** | Human observational component | EGFR T790M-mutant NSCLC receiving second-line osimertinib | n = 47 patients; CD68⁺NLRP3⁺ high/low groups, n = 24/23; CD68⁺IL-1β⁺ high/low groups, n = 24/23 | Osimertinib | Tumour-associated macrophages | NLRP3 and IL-1β expression | None | Higher macrophage NLRP3- and IL-1β-associated signals were associated with longer progression-free survival | A1 | B2 | Direct NSCLC evidence | Observational association; neither productive inflammasome activation nor NLRP3 dependence was demonstrated in the human component |
| **Feng et al., 2026 (39)** | Human transcriptomic/clinical-association component | GSE189357 scRNA-seq dataset and TCGA-LUAD cohort | Patient-level cohort size for these reused public-dataset analyses: NR in the primary article | Baseline LUAD context | Tumour-associated macrophage populations | UPP1 expression; *NLRP3*, *PYCARD*, *CASP1* and *GSDMD* transcript distributions; survival associations | None | UPP1 and inflammasome/pyroptosis-associated transcripts were enriched in macrophage populations, and UPP1 was associated with adverse clinical features | A1 | B2 | Direct NSCLC evidence | Public-dataset and observational analyses do not establish productive inflammasome activation or causal NLRP3 dependence |
| **Feng et al., 2026 (39)** | Macrophage mechanistic component | Human THP-1 macrophage model and murine BMDMs | n = 3 independent experiments for the NLRP3-related mechanistic assays | UPP1 overexpression | Macrophages | ASC oligomerisation; cleaved CASP1; GSDMD cleavage; LDH release; pyroptotic morphology; mature IL-1β secretion | shNLRP3; shGSDMD; CASP1 inhibitor VX-765; MitoTEMPO; cGAS inhibitor RU.521 | UPP1-associated mitochondrial stress/cGAS signalling induced NLRP3-dependent canonical inflammasome activation and pyroptotic output | A2/A3/A4/A5 | B1 | Direct NSCLC evidence | NLRP3 knockdown reduced IL-1β release and prevented UPP1-induced CASP1 and GSDMD cleavage, with independent ASC-assembly and CASP1-pathway support; the mechanistic experiments relied mainly on THP-1 cells and murine BMDMs rather than primary human TAMs |
| **Feng et al., 2026 (39)** | In vivo tumour/metastasis component | LLC + BMDM C57BL/6 co-injection model | n = 5 mice/group | Co-injection of LLC cells with Vector or *Upp1*-OE BMDMs | Macrophage–tumour compartment | Tumour growth; macrophage-associated GSDMD-N; tumour IL-1β; pulmonary metastatic burden | *Upp1* overexpression in co-injected macrophages | *Upp1*-OE macrophages were associated with increased primary tumour growth, inflammatory/pyroptotic signals and pulmonary metastatic burden | A5 | B2 | Direct NSCLC evidence | Gain-of-function co-injection model; NLRP3-specific perturbation was not performed within the in vivo component. |
| **Teng et al., 2020 (37)** | In vitro tumour-cell component | Human A549 and H1299 NSCLC cells | ≥3 independent biological experiments | Polyphyllin VI | Tumour cells | Cleaved CASP1; mature/cleaved IL-1β and IL-18; cytokine release; GSDMD cleavage; pyroptotic morphology/lysis | CASP1 inhibition and downstream pyroptosis-pathway manipulation | Polyphyllin VI induced inflammasome-associated and pyroptotic tumour-cell death | A3/A4/A5 | B2 | Direct NSCLC evidence | Downstream pathway perturbation does not establish NLRP3-specific causal dependence |
| **Teng et al., 2020 (37)** | In vivo xenograft component | A549 xenograft mouse model | n = 8 mice/group; five treatment groups | Polyphyllin VI | Tumour xenograft | Tumour inhibition; NLRP3; cleaved CASP1; cleaved IL-1β; cleaved GSDMD | Pharmacological treatment | Tumour suppression was accompanied by inflammasome- and pyroptosis-associated molecular changes | A3/A4/A5 | B2 | Direct NSCLC evidence | No NLRP3-specific genetic perturbation was performed |
| **Yuan et al., 2021 (45)** | In vitro tumour-cell component | Human A549 NSCLC cells | n ≥ 3 independent experiments where explicitly reported; otherwise NR | Cucurbitacin B | Tumour cells | NLRP3–NEK7 interaction; ASC specks; cleaved CASP1; mature IL-1β; GSDMD-N; pyroptotic morphology | TLR4 silencing; pharmacological pathway manipulation | Cucurbitacin B induced assembly-, CASP1-, cytokine-processing- and GSDMD-associated responses | A2/A3/A4/A5 | B2 | Direct NSCLC evidence | The principal genetic perturbation targeted TLR4 rather than NLRP3 |
| **Yuan et al., 2021 (45)** | In vivo syngeneic component | LLC-bearing C57BL/6J mice | n = 8 mice/group at treatment allocation | Cucurbitacin B | Tumour / tumour microenvironment | Tumour inhibition; NLRP3/GSDMD-associated pyroptotic phenotype | Pharmacological treatment; TLR4-centred pathway manipulation | Supports an in vivo TLR4/NLRP3-associated pyroptotic phenotype | A5 | B2 | Direct NSCLC evidence | NLRP3-specific genetic causality was not demonstrated |
| **Liu et al., 2025 (47)** | In vitro tumour-cell component | Human lung-cancer cell models | Biological replication: NR | Rabeprazole | Tumour cells | NLRP3; cleaved CASP1; GSDMD-related changes; LDH release; pyroptotic morphology | Rabeprazole; Z-LLSD-FMK | Rabeprazole induced an inflammasome/pyroptosis-associated inflammatory cell-death phenotype | A3 | B2 | Mechanistic extrapolation | Selective NLRP3 dependence and complete canonical pathway dependence were not demonstrated |
| **Liu et al., 2025 (47)** | In vivo component | Mouse lung-cancer model | Animal number per group: NR | Rabeprazole | Tumour | Tumour response; pyroptosis-associated markers | Pharmacological treatment | Supports in vivo relevance of the treatment-associated inflammatory cell-death phenotype | A3 | B2 | Mechanistic extrapolation | Model specificity and NLRP3-specific causality remain incomplete |
| **Ye et al., 2025 (51)** | Tumour-cell transcriptional-regulatory component | Human NSCLC cells | NLRP3 regulatory assays: n = 3 independent experiments; migration assays: n = 3; invasion assays: n = 5 where specified | NET exposure / MIR503HG–C/EBPβ manipulation | Tumour cells | NLRP3 mRNA/protein; C/EBPβ binding to the NLRP3 promoter | MIR503HG and C/EBPβ manipulation | Defines an NLRP3-associated transcriptional programme linked to metastatic behaviour | A1 | B2 | Direct NSCLC evidence | Regulation of NLRP3 expression does not establish inflammasome assembly, CASP1 activation or NLRP3-dependent canonical function |
| **Ye et al., 2025 (51)** | Human-tissue component | Paired NSCLC and adjacent non-tumour tissue | n = 17 paired samples | Baseline | Tumour tissue | MIR503HG/C/EBPβ/NLRP3-associated expression | None | Supports the clinical relevance of the transcriptional association | A1 | B2 | Direct NSCLC evidence | Correlative human evidence |
| **Ye et al., 2025 (51)** | In vivo metastasis component | Mouse NSCLC metastasis model | Animal number per group: NR | NET/MIR503HG/C/EBPβ manipulation | Tumour | Metastatic phenotype; NLRP3-associated regulation | MIR503HG/C/EBPβ manipulation | Supports an in vivo association between the regulatory axis and metastatic behaviour | A1 | B2 | Direct NSCLC evidence | Does not independently establish canonical NLRP3 inflammasome dependence |
| **De Zuani et al., 2024 (22)** | Candidate single-cell/spatial contextual evidence | Treatment-naïve human NSCLC | 25 treatment-naïve patients included overall (13 LUAD, 8 LUSC and 4 undetermined); Fig. 1 scRNA-seq overview, N = 24; spatial transcriptomics, n = 8 patients across 36 sections; 895,806 high-quality cells after QC | Baseline | Tumour, myeloid, lymphoid and stromal populations | Cell-type-resolved transcriptional states; macrophage reprogramming; spatial organisation; IL1B represented among myeloid transcriptional markers | None | Defines NSCLC macrophage states and spatial architecture relevant to subsequent investigation of inflammatory pathways | Not assigned | Not assigned | Indirect NSCLC evidence | NLRP3 was not directly interrogated as a study endpoint; although IL1B was represented among myeloid transcriptional markers, no NLRP3-focused transcriptional or spatial analysis, productive inflammasome activation, or NLRP3 dependence was demonstrated |
| **Sorin et al., 2023 (61)** | Candidate spatial immune-context evidence | Human LUAD | Discovery cohort: n = 416 patients; independent validation cohort: n = 60 | Baseline | Tumour and immune compartments | Single-cell spatial immune organisation | None | Defines spatially organised immune states and candidate inflammatory niches | Not assigned | Not assigned | Indirect NSCLC evidence | No direct NLRP3 interrogation |
| **Enfield et al., 2024 (63)** | Candidate spatial-architecture evidence | Treatment-naïve early-stage NSCLC | n = 81 patients; 198 samples; approximately 2.3 million cells profiled | Baseline | Myeloid and lymphoid populations | Spatial immune neighbourhoods | None | Defines myeloid–T-cell spatial architectures associated with immune evasion and clinical outcome | Not assigned | Not assigned | Indirect NSCLC evidence | No direct functional assessment of NLRP3 |
| **Wang et al., 2023 (64)** | Candidate spatial-transcriptomic progression context | Treatment-naïve LUAD | n = 5 patients | Baseline | Tumour, macrophage and stromal compartments | Spatial transcriptomics; histological-subtype-associated transcriptional states | None | Revealed spatial transcriptional heterogeneity and subtype-associated macrophage states during LUAD progression | Not assigned | Not assigned | Indirect NSCLC evidence | No direct NLRP3 interrogation |
| **Chen et al., 2023 (65)** | Candidate CAF/spatial context | Human NSCLC | scRNA-seq: n = 16 patients; spatial transcriptomics: n = 7 patients | Baseline | Cancer-associated fibroblasts, immune cells and tumour cells | POSTN⁺ CAF states; immune suppression; spatial cellular organisation | None | Identified POSTN⁺ CAF states associated with immune suppression and tumour progression | Not assigned | Not assigned | Indirect NSCLC evidence | No direct NLRP3 assessment |
| **Yan et al., 2025 (66)** | Treatment-associated multi-omic/spatial context | Human NSCLC before and after immuno-chemotherapy | n = 19 patients; 232,080 single-cell transcriptomes | Immuno-chemotherapy | Tumour cells; macrophages; CAFs; TLS-associated populations | Single-cell/spatial states; SPP1⁺ macrophage–COL11A1⁺ CAF interactions; TLS-associated programmes | None | Identified multicellular and spatial programmes associated with resistance to immuno-chemotherapy | Not assigned | Not assigned | Indirect NSCLC evidence | No direct NLRP3 interrogation |
| **Sandström Gerdtsson et al., 2023 (67)** | Candidate spatial immune-niche evidence | Human NSCLC | n = 33 NSCLC tumours | Baseline | Tumour and immune compartments | Spatial expression of 49 proteins; immune-infiltration niches | None | Defined phenotypically distinct spatial immune-infiltration niches | Not assigned | Not assigned | Indirect NSCLC evidence | No direct NLRP3 assessment |
| **Chen et al., 2024 (68)** | Candidate stem-immunity-hub evidence | Human pre-immunotherapy NSCLC | n = 68 patients; one pretreatment specimen per patient | Subsequent PD-1/PD-L1 blockade | T cells; dendritic cells; macrophages; fibroblasts | Multiplex RNA/protein imaging; stem-immunity hubs | None | Identified spatially organised stem-immunity hubs associated with subsequent immunotherapy response | Not assigned | Not assigned | Indirect NSCLC evidence | No direct NLRP3 interrogation |
| **Aung et al., 2025 (70)** | Treatment-associated spatial-context evidence | Advanced NSCLC treated with PD-1-based immunotherapy | n = 234 patients across three cohorts; spatial proteomics: n = 67; spatial compartment transcriptomics: n = 131 | PD-1-based immunotherapy | Tumour microenvironment | Spatial proteomic/transcriptomic response and resistance signatures | None | Defined clinically associated spatial signatures of immunotherapy outcome | Not assigned | Not assigned | Indirect NSCLC evidence | No direct functional assessment of NLRP3 activation or dependence |
| **Zhao et al., 2024 (71)** | KRAS-mutant spatial iTME contextual evidence | KRAS-mutant NSCLC treated with immune checkpoint inhibitors | n = 25 patients | Immune checkpoint inhibition | Tumour and immune compartments | Imaging mass cytometry; cellular neighbourhoods; spatial immune architecture | None | Identified spatial immune features associated with immunotherapy response and resistance | Not assigned | Not assigned | Indirect NSCLC evidence | No direct NLRP3 interrogation |
| **Kuhlmann-Hogan et al., 2024 (42)** | Murine macrophage metabolic-context component | EGFR-driven LUAD mouse models | Fig. 1: n = 3–10/group; Fig. 2: n = 3–13/group; Fig. 3: n = 4–21/group; Fig. 4: n = 3–12/group; Fig. 6: n = 5–14/group; ≥2–3 independent experiments depending on endpoint | Tumour-associated metabolic reprogramming | Alveolar macrophages | PPARγ-dependent lipid/cholesterol metabolism and macrophage functional states | Genetic/metabolic manipulation | Defines an LUAD-associated macrophage metabolic programme relevant to inflammatory competence | Not assigned | Not assigned | Indirect NSCLC evidence | NLRP3 expression, activation and dependence were not directly evaluated |
| **Kuhlmann-Hogan et al., 2024 (42)** | Human BALF contextual component | Human BALF samples | n = 10 donors: 5 lung-cancer cases and 5 non-lung-cancer disease controls | Clinical context | Alveolar macrophages | Macrophage metabolic phenotyping | None | Provides human contextual support for LUAD-associated macrophage-state observations | Not assigned | Not assigned | Indirect NSCLC evidence | No direct NLRP3 interrogation |
| **Zhi et al., 2026 (41)** | Human cryoablation-associated contextual component | Human NSCLC | Clinical comparison: n = 26 cryoablation and n = 52 thermal-ablation patients; scRNA-seq: n = 3 patients/group | Cryoablation versus thermal ablation | Macrophages / tumour immune compartment | cGAS–STING–CXCL10 signalling; CXCL10⁺ macrophage expansion; immune-remodelling signatures | Ablation modality | Cryoablation was associated with enhanced macrophage cGAS–STING–CXCL10 signalling and antitumour immunity | Not assigned | Not assigned | Direct NSCLC evidence | Demonstrates an alternative innate-immune pathway; NLRP3 activation or dependence was not demonstrated |
| **Zhi et al., 2026 (41)** | Experimental mechanistic component | Mouse/human NSCLC experimental systems | Animal group size: NR for the retained component unless explicitly specified in the corresponding primary figure legend | Cryoablation ± pathway manipulation / anti-PD-1 context | Macrophages | cGAS–STING activation; CXCL10 production; antitumour immune responses | Pathway manipulation | Provides functional support for a macrophage cGAS–STING–CXCL10 mechanism after cryoablation | Not assigned | Not assigned | Direct NSCLC evidence | Supports an alternative inflammatory mechanism rather than NLRP3 activation or dependence |
| **CANTOS exploratory lung-cancer analysis (24)** | Clinical downstream IL-1β-intervention evidence | Atherosclerosis population with exploratory lung-cancer outcomes | N = 10,061 randomised participants | Canakinumab | Systemic clinical intervention | Incident lung cancer; lung-cancer mortality | IL-1β neutralisation | Exploratory analyses reported lower lung-cancer incidence and mortality at selected canakinumab doses | Not assigned | Not assigned | Clinical downstream-intervention evidence | Not an NSCLC-specific treatment trial; lung-cancer outcomes were exploratory; downstream IL-1β blockade does not directly test NLRP3 inhibition |
| **CANOPY-1 (25)** | Clinical downstream IL-1β-intervention evidence | Phase III first-line advanced NSCLC | N = 643 randomised patients | Canakinumab + pembrolizumab + platinum-based chemotherapy versus placebo regimen | Clinical-trial population | PFS; OS | IL-1β blockade | Neither coprimary endpoint met its prespecified statistical criterion | Not assigned | Not assigned | Clinical downstream-intervention evidence | IL-1β blockade is not equivalent to direct NLRP3 inhibition |
| **CANOPY-A (26)** | Clinical downstream IL-1β-intervention evidence | Phase III completely resected NSCLC | N = 1,382 randomised patients | Adjuvant canakinumab versus placebo | Clinical-trial population | DFS; OS | IL-1β blockade | The primary DFS endpoint was not met; OS was not formally tested under the hierarchical strategy | Not assigned | Not assigned | Clinical downstream-intervention evidence | Does not directly test NLRP3 inhibition or dependence |
| **CANOPY-2 (27)** | Clinical downstream IL-1β-intervention evidence | Phase III previously treated advanced NSCLC | N = 237 randomised patients | Canakinumab + docetaxel versus placebo + docetaxel | Clinical-trial population | OS | IL-1β blockade | The primary OS endpoint was not met | Not assigned | Not assigned | Clinical downstream-intervention evidence | Does not establish NLRP3 pathway dependence |
| **CANOPY-N (28)** | Clinical downstream IL-1β-intervention evidence | Phase II resectable stage IB–IIIA NSCLC | N = 88: canakinumab n = 35; canakinumab + pembrolizumab n = 35; pembrolizumab n = 18 | Neoadjuvant canakinumab ± pembrolizumab | Clinical-trial population | MPR | IL-1β blockade | Neither canakinumab-containing arm met its prespecified proof-of-efficacy criterion | Not assigned | Not assigned | Clinical downstream-intervention evidence | Small treatment groups and imprecise estimates; downstream IL-1β blockade does not establish NLRP3 dependence |

**Note:** Evidence was mapped at the level of individual experimental evidence components rather than entire publications. Human observational analyses, in vitro mechanistic experiments and in vivo animal experiments were separated when they supported distinct conclusions. Sample size and biological replication are reported for each evidence component whenever explicitly stated in the primary article, Methods, supplementary material or figure legends. Where these data were not reported, the corresponding field is designated NR (not reported). NR denotes only that the relevant sample size or biological replication was not reported in the primary study and is not used as a substitute for heterogeneous or experiment-specific sample sizes. Technical replicates are distinguished from independent biological replicates where reported. The A-axis denotes the biological event directly assessed, whereas the B-axis denotes mechanistic attribution to NLRP3; the two axes are independent and do not constitute a hierarchy of evidentiary strength. B1 requires NLRP3-specific causal perturbation together with independent evidence supporting canonical inflammasome involvement; NLRP3 perturbation alone is insufficient for B1. B2 includes NLRP3-associated findings without demonstrated NLRP3 dependence, as well as NLRP3-dependent phenotypes for which canonical inflammasome involvement remains unresolved. B3 requires NLRP3 dependence together with evidence that canonical NLRP3–ASC/PYCARD–CASP1 signalling is dispensable. Evidence-source classification is independent of the A/B framework and describes disease and experimental context rather than mechanistic strength. Within the predefined eligibility scope and retrieval procedures described in Section 2.1 and Supplementary Table S2, Supplementary Table S1 includes all primary studies identified and retained for formal evidence evaluation, including supportive, alternative-mechanism, compartment-specific, treatment-associated, and spatial/contextual findings. Primary studies cited solely for general biological, clinical, technological, or methodological background were not included in the formal evidence map.

## 1.2 Supplementary Table S2

## Table S2. Literature search strategy and supplementary identification routes.

| **Search route** | **Database / source** | **Search strategy / identification approach** | **Records retrieved** | **Last search / identification date** |
| --- | --- | --- | --- | --- |
| Core NLRP3-focused search | PubMed | ("NLRP3"[Title/Abstract] OR "NLRP3 inflammasome"[Title/Abstract] OR "NOD-like receptor protein 3"[Title/Abstract]) AND ("NSCLC"[Title/Abstract] OR "non-small cell lung cancer"[Title/Abstract] OR "lung cancer"[Title/Abstract] OR "lung adenocarcinoma"[Title/Abstract]) AND ("activation"[Title/Abstract] OR "expression"[Title/Abstract] OR "priming"[Title/Abstract] OR "ASC"[Title/Abstract] OR "NEK7"[Title/Abstract] OR "caspase-1"[Title/Abstract] OR "CASP1"[Title/Abstract] OR "IL-1β"[Title/Abstract] OR "IL-18"[Title/Abstract] OR "gasdermin"[Title/Abstract] OR "GSDMD"[Title/Abstract] OR "pyroptosis"[Title/Abstract]) | 169 | August 20, 2026 |
| Core NLRP3-focused search | Web of Science | TS=("NLRP3" OR "NLRP3 inflammasome" OR "NOD-like receptor protein 3") AND TS=("NSCLC" OR "non-small cell lung cancer" OR "lung cancer" OR "lung adenocarcinoma") AND TS=("activation" OR "expression" OR "priming" OR "ASC" OR "NEK7" OR "caspase-1" OR "CASP1" OR "IL-1β" OR "IL-18" OR "gasdermin" OR "GSDMD" OR "pyroptosis") | 257 | August 20, 2026 |
| Core NLRP3-focused search | Europe PMC | TITLE_ABS:(NLRP3 OR "NLRP3 inflammasome" OR "NOD-like receptor protein 3") AND TITLE_ABS:(NSCLC OR "non-small cell lung cancer" OR "lung cancer" OR "lung adenocarcinoma") AND TITLE_ABS:(activation OR expression OR priming OR ASC OR NEK7 OR "caspase-1" OR CASP1 OR "IL-1β" OR "IL-18" OR gasdermin OR GSDMD OR pyroptosis) | 175 | August 20, 2026 |
| Supplementary spatial/context identification | Targeted database searching and citation tracking | NSCLC/lung-cancer terms were combined with concepts related to single-cell analysis, scRNA-seq, spatial transcriptomics, multiplex imaging and the tumour microenvironment to identify cellular and spatial studies that did not necessarily interrogate NLRP3 directly. Backward and forward citation tracking of relevant primary studies and reviews was also used. | NR | Up to August 20, 2026 |
| Supplementary downstream-intervention identification | Targeted database searching and citation tracking | IL-1β, interleukin-1 beta, canakinumab, CANTOS and CANOPY were examined in conjunction with lung-cancer/NSCLC concepts to identify downstream IL-1β intervention studies not necessarily retrieved by the NLRP3-focused core search. Citation tracking of relevant trial reports was also used. | NR | Up to August 20, 2026 |

**Note:** PubMed, Web of Science and Europe PMC were searched from database inception to August 20, 2026 for the core NLRP3-focused search. Searches were restricted to English-language publications. Because the core search required NLRP3/inflammasome terminology, supplementary identification routes were used to identify spatial/contextual NSCLC studies and downstream IL-1β intervention studies that might not directly mention NLRP3. Within the predefined eligibility scope, primary studies identified through the core search, supplementary identification routes and citation tracking were assessed for inclusion in the evidence map. Spatial, single-cell, transcriptomic or multiplex-imaging studies without direct NLRP3 interrogation were retained as contextual evidence rather than NLRP3-specific evidence unless an NLRP3-related finding was explicitly reported in the primary study. Reviews were used for background and citation tracking and were not entered as primary evidence-map components. NR, separate record count not documented for the supplementary identification route.

## 1.3 Supplementary Table S3

**Table S3. Comparison of the present evidence framework with previous lung-cancer and cancer-wide inflammasome reviews.**

| **Comparison domain** | **da Costa et al., 2023 (16)** | **Tengesdal et al., 2023 (15)** | **Shadab et al., 2023 (49)** | **Present Review** |
| --- | --- | --- | --- | --- |
| **Primary scope** | Inflammasome-driven inflammation in lung cancer, with particular emphasis on NSCLC | NLRP3 biology and therapeutic opportunities across cancer types | Divergent functions of the NLRP3 inflammasome across cancer settings | NLRP3-associated biology in NSCLC, with explicit separation of biological readout, mechanistic attribution and evidence context |
| **Principal organising focus** | Inflammasome involvement across lung-cancer initiation, progression, epithelial–mesenchymal transition, metastasis and therapy resistance | NLRP3 activation in cancer, IL-1-mediated inflammation and opportunities for pharmacological NLRP3 inhibition | Context-dependent tumour-promoting and tumour-restraining functions of NLRP3 in cancer | Experiment-level evidence interpretation using independent A-axis, B-axis and evidence-source dimensions |
| **Expression/priming distinguished from productive inflammasome activation** | Biological mechanisms are discussed, but not organised using a formal experiment-level readout classification | NLRP3 activation mechanisms are discussed, but not classified using the A1–A5 experiment-level scheme applied here | NLRP3 activation and downstream signalling are discussed biologically, but not organised using the present formal experiment-level scheme | Formally separated through the A1–A5 biological-event framework |
| **Assembly, CASP1 activation, mature IL-1β/IL-18 production and pyroptotic execution treated as distinct evidence components** | Discussed where relevant, without an equivalent formal categorical framework | Mechanistically discussed, without the present experiment-level categorical separation | Mechanistically discussed, without the present experiment-level categorical separation | Treated as experimentally distinct A-axis readouts rather than interchangeable evidence of pathway activation |
| **NLRP3-specific mechanistic dependence distinguished from association** | Mechanistic evidence is discussed, but no equivalent formal dependence axis is applied across individual evidence components | NLRP3-dependent mechanisms and pharmacological targeting are discussed, but not using the B1–B3 classification applied here | Mechanistic and context-dependent functions are discussed, but not using the present formal dependence classification | Formally separated through B1–B3 according to the degree and type of NLRP3-specific mechanistic attribution |
| **Evidence source/disease relevance classified independently from mechanistic strength** | Lung-cancer evidence is synthesised by biological role; no equivalent formal source-classification axis is used | Cancer-wide mechanistic and therapeutic evidence is synthesised; no NSCLC-specific source-classification axis is used | Cancer-wide evidence is synthesised across tumour contexts; no NSCLC-specific source-classification axis is used | Direct NSCLC evidence, Indirect NSCLC evidence, Mechanistic extrapolation and Clinical downstream-intervention evidence are classified independently from A/B assignment |
| **Cellular source explicitly retained in evidence interpretation** | Cellular contexts are discussed where relevant | Tumour- and immune-cell mechanisms are discussed | Cellular context is considered in the discussion of divergent NLRP3 functions | Cellular source is retained as an independent interpretative variable |
| **Treatment context explicitly retained in evidence interpretation** | Therapy resistance and therapeutic implications are discussed | Therapeutic targeting and treatment-related implications are discussed extensively | Treatment-related functions are discussed across cancer settings | Untreated, treatment-conditioned and on-treatment evidence are distinguished where applicable |
| **Human observational and mechanistic experimental evidence within the same publication classified separately** | No equivalent formal experiment-component assignment rule | No equivalent formal experiment-component assignment rule | No equivalent formal experiment-component assignment rule | Separate evidence components may receive different A/B assignments according to the experiments actually performed |
| **Treatment of spatial/transcriptomic evidence** | Not a formal evidence-classification dimension | Not a formal evidence-classification dimension | Not a formal evidence-classification dimension | Retained as contextual or Indirect NSCLC evidence unless functional NLRP3 activity and pathway dependence are directly demonstrated |
| **Treatment of downstream IL-1β intervention evidence** | Therapeutic implications of inflammasome-related inflammation are discussed | IL-1 blockade and NLRP3-targeted therapy are discussed as related but biologically distinct strategies | Therapeutic targeting of NLRP3-associated inflammation is discussed | Classified separately as Clinical downstream-intervention evidence and not interpreted as direct clinical testing of NLRP3 inhibition |
| **Unit of formal classification** | Narrative synthesis at the study/topic level | Narrative mechanistic and therapeutic synthesis | Narrative synthesis across cancer contexts | Individual experimental evidence component |
| **Intended role of the framework** | Biological synthesis of inflammasome involvement in lung cancer | Mechanistic and therapeutic synthesis of NLRP3 in cancer | Synthesis of context-dependent NLRP3 functions in cancer | Organisational and interpretative framework intended to reduce over-inference across biological readouts, mechanistic attribution and evidence context |

**Note:** This comparison concerns the organisation and interpretation of evidence rather than the overall quality, scope or comprehensiveness of the cited reviews. “No equivalent formal classification” indicates that a previous review did not apply the same experiment-level categorical framework used in the present Review; it does not imply that the underlying biological distinction was absent from, or unrecognised by, the previous literature. The present framework is intended as an organisational and interpretative tool rather than a claim that the biological relationship between inflammasome/NLRP3 signalling and lung cancer is newly recognised.

## 1.4 Supplementary Table S4

**Table S4. Quantitative summary of IL-1β-targeted clinical trials relevant to lung cancer and NSCLC: distinction from direct NLRP3 inhibition.**

| **Trial** | **Phase/design and population** | **Intervention and sample size** | **Prespecified primary endpoint** | **Main effect estimate (95% CI)** | **Multiplicity/statistical status** | **Registration** | **Principal safety findings** | **Interpretation for NLRP3 biology** |
| --- | --- | --- | --- | --- | --- | --- | --- | --- |
| CANTOS (24) | Randomised, double-blind, placebo-controlled cardiovascular outcomes trial; patients with previous myocardial infarction and persistent inflammation (hsCRP ≥2 mg/L); not an NSCLC treatment trial | Canakinumab (50, 150, or 300 mg subcutaneously every 3 months) versus placebo; N=10,061 | Parent trial: recurrent cardiovascular events (nonfatal myocardial infarction, nonfatal stroke, or cardiovascular death) | Exploratory lung-cancer outcomes: incident lung cancer HR 0.61 (95% CI 0.39–0.97) for 150 mg and HR 0.33 (95% CI 0.18–0.59) for 300 mg; lung-cancer mortality HR 0.23 (95% CI 0.10–0.54) for 300 mg | Lung-cancer analyses were exploratory and hypothesis-generating rather than prespecified confirmatory oncology endpoints | NCT01327846 | Fatal infection or sepsis occurred more frequently with canakinumab than placebo | Downstream IL-1β intervention; exploratory relevance to lung cancer; not a direct test of NLRP3 inhibition or NLRP3 dependence |
| CANOPY-1 (25) | Phase III, randomised, double-blind, placebo-controlled trial; previously untreated advanced/metastatic NSCLC without EGFR or ALK alterations | Canakinumab + pembrolizumab + platinum-based chemotherapy versus placebo + pembrolizumab + chemotherapy; N=643 | Coprimary endpoints: progression-free survival (PFS) and overall survival (OS) | PFS: HR 0.85 (95% CI 0.67–1.09), P=.102; OS: HR 0.87 (95% CI 0.70–1.10), P=.123 | Coprimary efficacy endpoints with prespecified statistical criteria; neither endpoint met its criterion | NCT03631199 | No unexpected safety signal; infection rates were comparable; some laboratory abnormalities, including neutropenia and ALT elevation, were more frequent with canakinumab | Downstream IL-1β blockade; not a direct test of NLRP3 inhibition |
| CANOPY-A （26） | Phase III, randomised, double-blind, placebo-controlled trial; completely resected stage II–III NSCLC after adjuvant cisplatin-based chemotherapy | Canakinumab versus placebo; N=1,382 | Disease-free survival (DFS) | Median DFS: 35.0 versus 29.7 months; HR 0.94 (95% CI 0.78–1.14); one-sided P=.258 | Hierarchical testing strategy; OS was not formally tested because the primary DFS endpoint was not statistically significant | NCT03447769 | Grade ≥3 adverse events: 20.8% versus 19.6%; treatment discontinuation due to adverse events: 4.3% versus 4.1%; no new safety signal identified | Downstream IL-1β blockade; not a direct test of NLRP3 inhibition |
| CANOPY-2 （27） | Phase III, randomised, double-blind, placebo-controlled trial; previously treated advanced NSCLC after platinum-based chemotherapy and PD-(L)1 therapy | Canakinumab + docetaxel versus placebo + docetaxel; N=237 | Overall survival (OS) | Median OS: 10.6 versus 11.3 months; HR 1.06 (95% CI 0.76–1.48); one-sided P=.633 | OS was the primary efficacy endpoint; exploratory subgroup analyses were not designed to establish confirmatory treatment effects | NCT03626545 | Grade 3–4 adverse events: 62% versus 64%; grade 5 adverse events: 8% versus 5% | Downstream IL-1β blockade; not a direct test of NLRP3 inhibition |
| CANOPY-N (28) | Phase II, randomised, open-label neoadjuvant trial; resectable stage IB–IIIA NSCLC | Canakinumab alone (n=35), canakinumab + pembrolizumab (n=35), pembrolizumab (n=18); N=88 | Major pathological response (MPR) by central pathology assessment in canakinumab-containing arms | MPR by central pathology assessment: canakinumab 2.9% (95% CI 0.07–14.92); canakinumab + pembrolizumab 17.1% (95% CI 6.56–33.65) | Prespecified arm-specific posterior probability criteria for proof of efficacy in canakinumab-containing arms; neither criterion was met | NCT03968419 | No unexpected safety signal; 84/88 patients underwent surgery and 72/84 underwent surgery within the prespecified 6-week window | Downstream IL-1β blockade; small treatment groups and imprecise estimates; not a direct test of NLRP3 inhibition |

**Note:** Canakinumab is an IL-1β-neutralising monoclonal antibody and does not directly inhibit NLRP3. These trials therefore inform downstream IL-1β biology rather than direct NLRP3 target validation. CANTOS lung-cancer outcomes were exploratory analyses conducted within a cardiovascular trial rather than a lung-cancer treatment population. Effect estimates and 95% confidence intervals are presented to distinguish failure to demonstrate benefit from limited statistical precision. Biomarker and subgroup analyses are not interpreted as confirmatory evidence of treatment benefit or NLRP3 dependence.
